# Conceptual environmental impact assessment of a novel self-sustained sanitation system incorporating a quantitative microbial risk assessment approach

**DOI:** 10.1016/j.scitotenv.2018.05.062

**Published:** 2018-10-15

**Authors:** Aikaterini Anastasopoulou, Athanasios Kolios, Tosin Somorin, Ayodeji Sowale, Ying Jiang, Beatriz Fidalgo, Alison Parker, Leon Williams, Matt Collins, Ewan McAdam, Sean Tyrrel

**Affiliations:** School of Water, Energy and Environment, Cranfield University, MK43 0AL, UK

**Keywords:** Nano-membrane toilet, Conventional sanitation systems, Environmental assessment, Quantitative microbial risk assessment

## Abstract

In many developing countries, including South Africa, water scarcity has resulted in poor sanitation practices. The majority of the sanitation infrastructures in those regions fail to meet basic hygienic standards. This along with the lack of proper sewage/wastewater infrastructure creates significant environmental and public health concerns. A self-sustained, waterless “Nano Membrane Toilet” (NMT) design was proposed as a result of the “Reinvent the Toilet Challenge” funded by the Bill and Melinda Gates Foundation. A “cradle-to-grave” life cycle assessment (LCA) approach was adopted to study the use of NMT in comparison with conventional pour flush toilet (PFT) and urine-diverting dry toilet (UDDT). All three scenarios were applied in the context of South Africa. In addition, a Quantitative Microbial Risk Assessment (QMRA) was used to reflect the impact of the pathogen risk on human health. LCA study showed that UDDT had the best environmental performance, followed by NMT and PFT systems for all impact categories investigated including human health, resource and ecosystem. This was mainly due to the environmental credits associated with the use of urine and compost as fertilizers. However, with the incorporation of the pathogen impact into the human health impact category, the NMT had a significant better performance than the PFT and UDDT systems, which exhibited an impact category value 4E + 04 and 4E + 03 times higher, respectively. Sensitivity analysis identified that the use of ash as fertilizer, electricity generation and the reduction of NOx emissions were the key areas that influenced significantly the environmental performance of the NMT system.

## Highlights

• Detailed LCA study is conducted for novel toilet system, Nano Membrane Toilet (NMT).
• Pour Flush Toilet (PFT), Urine Diverting Dry Toilet (UDDT) and the NMT are assessed.
• LCA study has been coupled with QMRA analysis for Human Health Impact Category.
• Under traditional LCA, the UDDT demonstrates a better environmental performance.
• Incorporating QMRA the NMT system performs better against the human health impact.

## Graphical abstract

**Figure:**
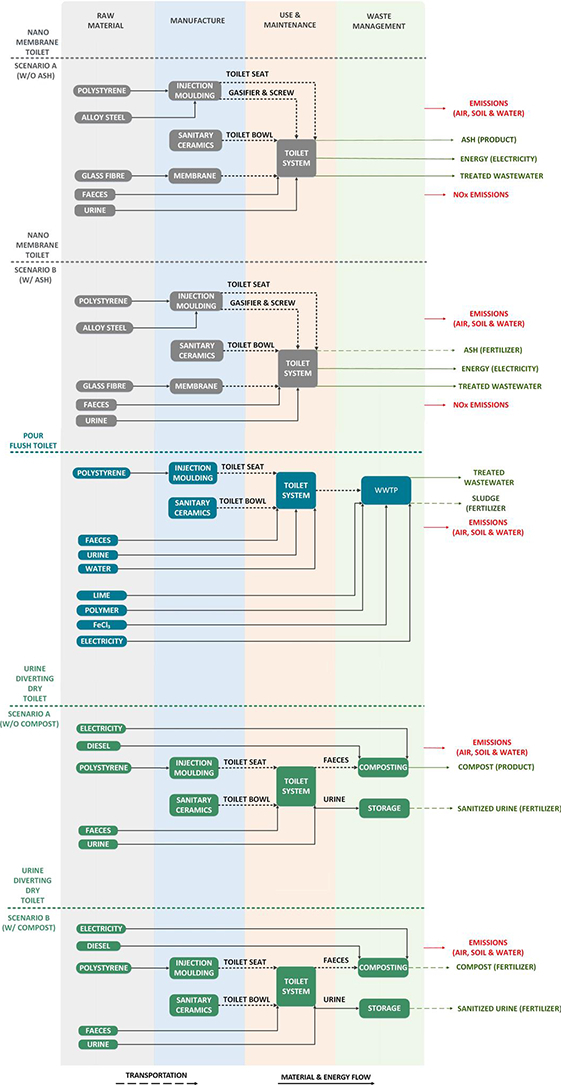

## 1 Introduction

Provision of sanitation facilities which meet the international baseline standards (UNICEF and WHO, 2017), constitutes a major problem in developing world. In particular, in sub-Saharan African countries only 28% of the population were reported to have access to at least a basic sanitation service in 2015 (UNICEF and WHO, 2017). This situation is compounded by the lack of proper sewerage and the poor operation and maintenance of the domestic faecal sludge management facilities (Wang et al., 2014). Existing sanitation practices in the majority of developing countries rely mainly on on-site waste treatment approaches (African Water Facility, 2014; Nyenje et al., 2010; Wang et al., 2014), including flush and waterless latrines connected to pit or septic tanks as a basic treatment of the waste (Huuhtanen and Laukkanen, 2009; Kjellén et al., 2011). Depending on the deployment, the waste can be a sludge with mixed urine and faeces, or source separated urine and faeces. In the case of faecal sludge, on-site treatment involves mainly solid-liquid separation by sedimentation in the septic tank and the subsequent filtration of the effluent into the ground (Brikké and Bredero, 2003; Orner and Mihelcic, 2018; Tilley et al., 2014). The remaining solids are degraded under anaerobic conditions for a period of 6 months to 10 years to produce a nutrient-rich humus (Schönning et al., 2005). Source separation of urine can be achieved by waterless systems through a specific user interface design. In this case, urine is sanitized in a storage tank and faeces are composted in a dehydration vault for a minimum period of 6 months (Tilley et al., 2014). Based on the scientific literature, after the treatment period both products can be used as organic fertilizers in local fields, provided that proper sanitization is attained (Andersson, 2015; Karak and Bhattacharyya, 2011; Kirchmann and Pettersson, 1995; Petersens and Beck-friis, 2012). However, although the social acceptability of their use in agriculture varies considerably among the developing countries (Moilwa, 2007; Mugivhisa, 2015), in this environmental study the given products have been considered scientifically acceptable, similarly to other relevant LCA studies (Kulak et al., 2017; Remy and Jekel, 2008; Flores et al., 2009).

Although, these conventional sanitation methods have been established in developing countries for many years, in practice they often fail to meet the design standards and operation requirements recommended by WHO. As a result, they pose significant human health risks and environmental concerns. To exemplify, a sanitation sustainability survey conducted in South Africa showed that 28% of the examined toilet systems was inadequately functional, while negligence of proper maintenance and operation of the pits was generally observed (Dwaf, 2012). According to another study that examined the challenges linked to the provision of a sustainable sanitation in Kigali city in Rwanda, odour and insect issues accounted for the second and fourth most common problems faced during the use of existing sanitation systems (Tsinda et al., 2013), and the difficulty in cleaning the toilet facility was perceived as the third major concern. In addition, there has been evidence that improper design and use of the pit latrines, due to both human and environmental factors, can facilitate the transmission of pathogens to the groundwater (Graham and Polizzotto, 2013; Nyenje et al., 2013; Pickering et al., 2012; Stenström et al., 2011). As the importance of a safely managed sanitation system has been highly emphasized as a prerequisite of social and economic welfare (UNICEF and WHO, 2017), such indications need to be taken into earnest consideration and measures to improve existing sanitation, waste and wastewater management infrastructures in developing countries need to be deployed.

A novel approach towards the provision of a sustainable sanitation in the developing countries has been proposed by Cranfield University in the context of the Bill & Melinda Gates Foundation's “Reinvent the Toilet Challenge” (Hanak et al., 2016; Onabanjo et al., 2017, Onabanjo et al., 2016a, Onabanjo et al., 2016b). The design and operating principle of the waterless Nano Membrane Toilet (NMT), is to incorporate into a single system the on-site combustion of human faeces and the purification of urine by membrane separation. The system benefits from the safe in-situ waste management which generates clean water and energy as valuable by-products, with the latter being recovered for meeting household power needs. The NMT technology is in its early stage of development and its efficacy at an end-user level has not yet been fully assessed. However, proceeding at this stage, with an *ex-ante* environmental appraisal of the system life cycle is likely to yield critical insights into its relative performance compared to established technologies and, in turn, into the potential areas of further optimization.

The majority of the life cycle assessment studies on sanitation technologies reported in literature explore primarily the environmental performance of the waste and wastewater treatment techniques employed in different toilet systems, excluding the life cycle of the latter systems (Flores et al., 2009; Friedrich et al., 2009; Remy and Jekel, 2007; Roux et al., 2011; Thibodeau et al., 2014). More precisely, Benetto et al. have carried out a comparative life cycle assessment (LCA) study of the EcoSan (Ecological Sanitation) concept, and the conventional wastewater treatment facilities for the case of Luxembourg (Benetto et al., 2009). Results have demonstrated an outperformance of the EcoSan system over small-scale conventional plants. Remy and Jerel, (Remy and Jekel, 2008) have evaluated the environmental impact of source separation sanitation systems and conventional sanitation systems, i.e. connected to sewage treatment plant, in the context of an urban settlement in Germany. Based on their research findings, certain source separation methods manifest a better profile over the conventional wastewater treatment for selected impact categories. Only a few studies have assessed the environmental impact of different toilet systems -flush, composting, pit latrine and source-separating toilets-, either on a standalone basis (Kohler Co, 2014) or along with the involved waste and/or wastewater activities (Anand and Apul, 2011; Devkota et al., 2013; Gao et al., 2015; Kulak et al., 2017). Although the aforementioned studies provide important views on the environmental performance and relative competitiveness of existing sanitation alternatives, a new approach to the life cycle assessment of such systems is proposed by incorporating the health risks linked to pathogen exposure. To elaborate, the results of the quantitative microbial risk analysis (QMRA), widely employed in the appraisal of drinking water quality and the respective health risks (Fuhrimann et al., 2016; Petterson and Ashbolt, 2016; WHO, 2016), have been coupled with the LCA results in order to incorporate the pathogen risk into the environmental impact of wastewater management systems on human health (Dong et al., 2017; Harder et al., 2016, Harder et al., 2015, Harder et al., 2014; Kobayashi et al., 2015). Such holistic approach has not been yet applied in the environmental assessment of the contemporary sanitation systems. This knowledge gap is likely to be filled by the present research work which aims at providing a comprehensive environmental assessment of the Nano Membrane Toilet (NMT) against the established on-site unsewered sanitation technologies in the context of South Africa, with the view to identifying areas of potential improvement of the NMT system which is still in the development phase. The conventional technologies selected for this study are the pour flush and the urine-diverting-dry toilet systems. In order to evaluate the given technologies on a fair basis, the conventional ones have been considered to be connected with a (semi)-centralized waste and wastewater treatment facility to ensure the same quality of provided service as to that of the NMT system. However, an important aspect to be mentioned is that the evaluation of the different sanitation systems as employed in this study, facilitates primarily the exploration of the critical environmental aspects of the NMT system rather than the rigorous comparison of the selected technologies, as their applicability in the broader context of developing countries is highly dependent not only on environmental, but also on other important factors, like economic and societal as well as the deployment context.

## 2 Methodology

In the frame of this environmental study, the performance of the selected sanitation systems has been evaluated, firstly, according to the traditional LCA methodology and, secondly, based on the aggregation of the QMRA and LCA results for the human health impact category. In the absence of representative input data covering all major cities of South Africa, indicative data on transportation distance and operating efficiency of the studied waste and wastewater treatment plants have been employed for the city of Alice, located at the Eastern Cape Province of South Africa (Iweriebor et al., 2015; Mnkeni, 2009; Momba et al., 2009; Sibanda and Okoh, 2012).

### 2.1 Life cycle assessment

#### 2.1.1 Goal and scope

The goal of this LCA study is to evaluate the environmental performance of the novel NMT system and that of the conventional sanitation systems most commonly seen in developing countries, i.e. pour flush and urine diverting dry toilets (Rieck et al., 2013; African Water Facility, 2014). The effect of the unit processes on the environmental footprint of each examined system is investigated with the view to identifying areas of potential improvement of the NMT design and operation. The LCA modelling has been carried out in SimaPro 8.0 software. The scope of this study includes the processes involved in the manufacture of the selected toilet systems and the safe treatment of the human waste as handled from the specific sanitation technologies. The functional unit has been selected as “the provision of a sanitation service for the daily defecation of a 10-adult occupant household in South Africa”, as dictated by the NMT-Project. The reference flow has been set to 2 kg human faeces (Hanak et al., 2016) and 14.2 kg urine (Rose et al., 2015).

#### 2.1.2 System boundaries

The system boundaries of the sanitation systems examined in this LCA study have been defined based on a “cradle-to-grave” approach, which considers the material and energy flows and the associated emissions from the raw material extraction until the disposal of an end product or service (Ayres, 1995). The end of life of the infrastructure involved in the provided service has been excluded in all three systems. The operating principle and process units involved in the life cycle stages, which are particular for each sanitation technology, are described below.

### 2.2 Nano membrane toilet (NMT) system

The NMT system is a waterless sanitation system, developed at Cranfield University, which enables the separation of the excreta through sedimentation. The separated faeces are first dried and transferred through a mechanical screw to a combustor for their conversion to energy and ash. The urine is preheated with the aid of the flue gas generated from the faeces combustion, and is driven to a nanostructured membrane, where separation of the unbound water is attained. The system is capable of delivering treated wastewater and excess amount of energy, which can be used in the form of electricity for household needs. A comprehensive description of the NMT system is provided in the referenced research studies (Hanak et al., 2016; Onabanjo et al., 2016a; Kolios et al., 2018; Jurado et al., 2018).

**Fig. 1:**
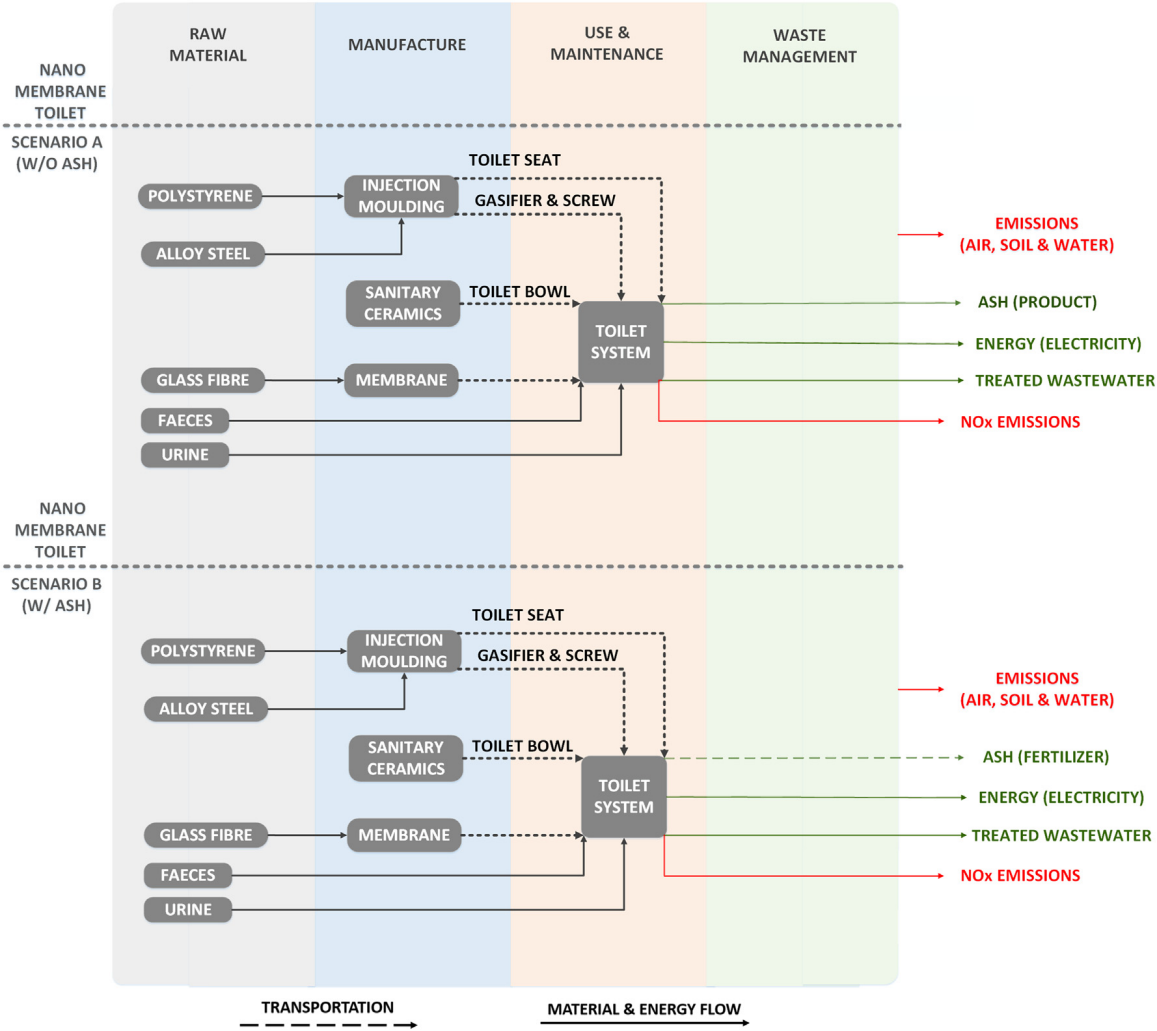
System boundaries of the Nano Membrane Toilet (NMT) for Scenario A and B.

Though the design of the toilet framework is not completed yet, materials for its construction have been selected, at this stage, based on information provided by the NMT project. The major materials employed are plastic for the toilet seat and ceramic for the toilet bowl. However, as the design of the NMT system is not the same as that of a conventional toilet (The Nano Membrane Toilet, n.d.), in order to compensate in terms of material input for the space required to accommodate the internal system components -membrane, combustor screw and etc.-, the use of a cistern has been considered. In addition to this consideration, 20% additional input material based on the material requirements of a conventional toilet system (Genty et al., 2014), has been employed for the construction of the toilet bowl, seat and cistern. As shown in Fig. 1, the system boundaries of the NMT include the use of polystyrene for the toilet seat and cistern construction through the process of injection moulding and the use of sanitary ceramics for the toilet bowl. The membrane has been assumed to be manufactured by glass fibre, while the combustor and the screw conveyor by alloy steel undergoing injection moulding.

In terms of the use phase of the NMT system, the system generates energy, which in this LCA study is expressed as electricity, NOx emissions and ash from the combustion of faeces with the latter one being disposed. Additionally, treated wastewater is produced from the urine purification through membrane filtration which can be used for household reuse purposes, such as garden irrigation. It is important to be mentioned that this water could be potentially used for other domestic cleaning purposes as well, but the aspect of social acceptability of the practice remains to be considered. The environmental benefits associated with the production of electricity and treated wastewater have been reflected in the LCA study by substituting the respective amount of the generated products with that deriving from the conventional tap water production and the market of medium voltage electricity generation. Moreover, as the NMT system is under development and the theoretical potentiality of the generated products has not been yet evaluated, two scenarios have been developed with respect to the use of ash as fertilizer substitute. The scenario A (w/o ash) considers the ash as a product of no practical use which is disposed to the environment without any specific harm. On the other hand, the scenario B (w/ ash) considers the fertilizer value of the ash based on reported literature which highlights the nutrient value of ash produced from biomass combustion and its fertilizer properties (Hatfield and Stewart, 1997; Pels et al., 2005; Schiemenz and Eichler-Löbermann, 2010). In this scenario, transportation of the ash from the set deployment location, city of Alice, to a local field in Blinkwater of Eastern Cape, at a distance of 34 km, has been considered.

The maintenance of the system involves normally the cleaning of the membrane every two months, however, for simplification purposes this requirement has been replaced in this study with the need of a new membrane every two months and has been modelled with the input material of “glass fibre”. The particular maintenance schedule has been determined based on a conservative approach driven by preliminary research findings. The lifetime of the NMT system has been set to 7 years, as dictated by the NMT-Project. This is a typical value of the lifetime of domestic appliances in most of the developing countries, including South Africa. Moreover, considering the cost of maintenance service required for the NMT system which involves the replacement of high capital components by specialist technicians, the selected value of lifetime renders the NMT an economically feasible sanitation system.

### 2.3 Pour flush toilet (PFT) system

In this toilet system, the excreta is flushed away with water and collected in a cesspit. Blackwater is transported to a pump station and, thereafter, directed to a sewage treatment plant by tankers. Upon completion of the wastewater treatment, safe disposable water and sludge are generated, with the latter being suitable for farming applications.

**Fig. 2:**
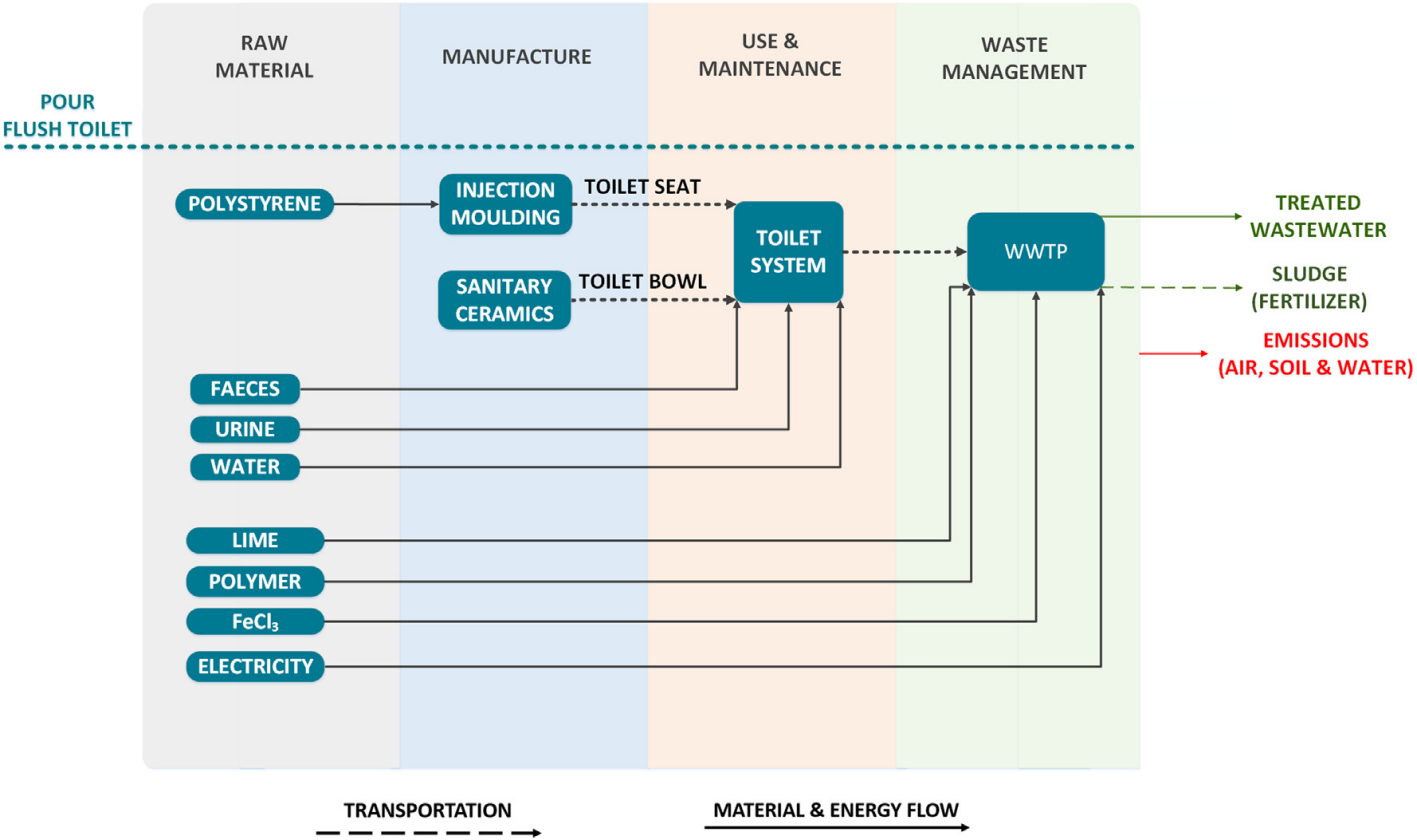
System boundaries of the Pour Flush Toilet (PFT).

As shown in Fig. 2, the raw material extraction and manufacture steps of the PFT system involve the same type of materials and processes for the toilet seat and bowl manufacture as presented for the NMT. The use phase entails the consumption of water which has been defined to be 150 l for the 10-occupant household – 5 flushes per day per capita (Viljoen, 2015) and 3 l of water per flush (Nyarko et al., n.d.). Since the lifetime of toilet systems has been reported in the range of 12.5 to 17.5 years (Gandy, 2011), the lifetime of the PFT system has been assumed to be 15 years.

With respect to the treatment of the blackwater, the wastewater treatment plant (WWTP) located at Fort Beaufort in the Eastern Cape Province of South Africa has been selected at an approximate distance of 23 km from the city of Alice. The assumed WWTP employs the activated sludge technology and based on that the required energy and material input for the operation of the plant has been adopted from literature (Lassaux et al., 2007). The treated water has been considered to be discharged to a local river and the generated sludge has been considered as fertilizer for farming. In the latter application, the nutrient content of the human urine has been assumed to be incorporated in the produced sludge, to reflect potential credits linked to its use as fertilizer. The sludge from the WWTP has been assumed to be applied as fertilizer to the local field, mentioned in the case of the NMT system, located 13 km away from the plant.

### 2.4 Urine diverting dry toilet (UDDT) system

The UDDT system operates without the use of water and allows the separation of the urine and faeces through a uniquely designed user interface. Urine is collected in a cesspit for storage and a later use as fertilizer. There are different methods available for the faeces management (Rieck et al., 2013); in this study the use of a single vault with interchangeable containers has been considered.

**Fig. 3:**
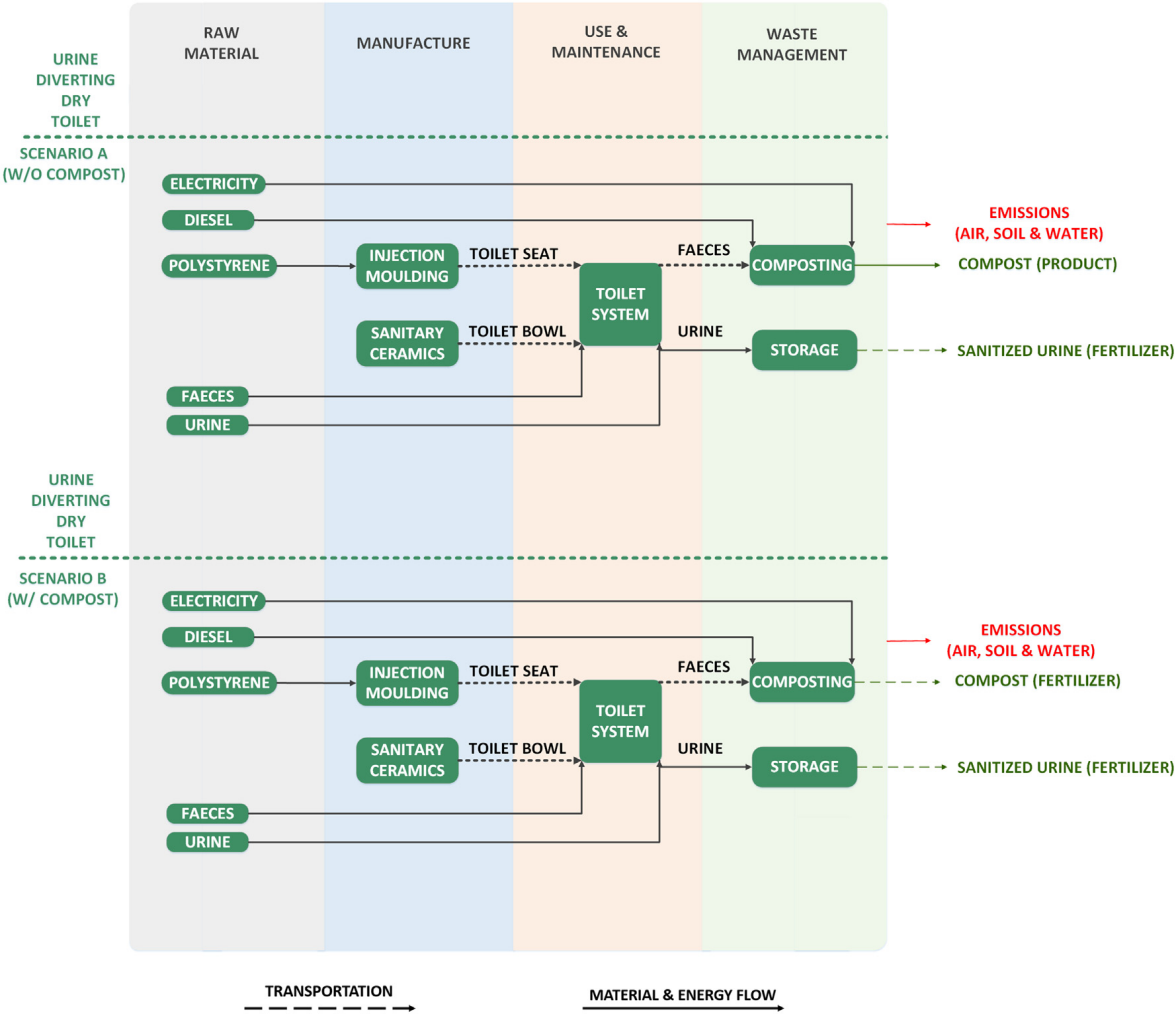
System boundaries of the Urine Diverting Dry Toilet (UDDT) for Scenario A and B.

For the construction of the given sanitation system, the same approach to that of the PFT system has been adopted for the raw material extraction and manufacture stages as depicted in Fig. 3. A windrow facility has been assumed to be located at Fort Beaufort, at a 23 km distance from the city of Alice, where the co-composting of the faecal matter with other organic material takes place. Though co-composting plant involves the mixing of human faeces with other organic matter, the production of the organic material has been excluded from the system boundaries, as the scope of this LCA involves only the material and energy input and output related to the treatment of the human waste generated from the examined household as defined in the functional unit. The role of the organic matter is only complimentary to ensure the proper C:N ratio for an efficient composting. The material and energy requirements for the treatment of faeces have been solely quantitatively estimated based on similar composting process for 1 ton of treated “feedstock” material (Department of Environment and Conservation NSW, 2006; van Haaren, 2009). The leachate produced during the composting process has been assumed to be recycled back to control the moisture of the composting matter (David Border Composting Consultancy, 2002), excluding the energy and material input involved in its collection from the system boundaries. Since the use of faecal compost has not been yet employed in agriculture to such a considerable extent in the developing countries, two scenarios have been also explored in this case. Scenario A (w/o compost) considers the compost as a disposable product with no fertilizer value and no environmental impact, whereas the scenario B (w/ compost) takes into account the potential use of the compost as a fertilizer to a local field (theoretical potential). The nutrient content, as non-available form of fertilizer, in the leachate mentioned above has been extracted from literature for a composting process of other similar organic matter and been estimated as 2.6%, 1.7% and 8.2% of the initial N, P and K content (Sommer, 2001). The treated compost is transported from the composting plant to the same local field in Blinkwater, as in the case of the NMT system, located at a distance of 13 km, whereas the urine solution stored in the cesspit is transferred to the same field at an approximate distance of 34 km. Lastly, similarly to the PFT system, the lifetime of the UDDT system has been defined also to 15 years.

### 2.5 Common assumptions

Some common assumptions employed for all sanitation systems include the transportation of the toilet system from the sanitary-ware factory located at Krugersdorp, in the Gauteng Province of South Africa to the city of Alice, in the Eastern Cape Province of South Africa, at a distance 920 km, where the sanitation systems are assumed to be based. Moreover, the energy and material requirements for the assembly of the toilet systems have been excluded due to the absence of coherent literature data. The construction of the toilet superstructure, the hand wash facility and the use of any cover material and toilet paper has been excluded from this study according to the LCA methodology (European Commission, 2010), as they have been assumed to be the same in all examined systems. The construction of the cesspits and the required pipelines connected to them, as well as the infrastructure of the waste and wastewater treatment plants, discussed below, have also been omitted as their respective contribution to the LCA has been proven negligible (Anand and Apul, 2011; Remy and Jekel, 2007).

In terms of the conventional sanitation systems, PFT and UDDT, the assumption of emptying the cesspits on a daily basis has been adopted. Although in reality the specific process takes place every 8–10 months (Cleansing Service Group Ltd, 2013), the assumption considered in this LCA study yields the same environmental impact as in the former case. To elaborate, in the LCA software the estimation of the environmental impact of transporting a material is merely based on the distance and the mass transported. Thus, environmental impact of transporting the amount of human waste generated over that time period -8 to 10 months- to the waste treatment plant needs to be amortized per day, since the functional unit refers to the specific amount of human waste produced on a daily basis. This impact, in absolute value, is the same as what it is being estimated in the LCA model by considering the daily transportation of the human waste. In addition to the aforementioned assumption, the energy and material requirements of the given process have been excluded. The transportation of the faecal or excreta matter from the location of the sanitation systems to the selected waste and wastewater treatment facilities has been incorporated in the system boundaries. Moreover, fertilizer substitution from the products generated from the treatment plants has been estimated based on certain mineralization factors for their N, P and K content, as mentioned in Table 1. The transportation of the potential fertilizer products from the waste and wastewater plants to a local field has also been incorporated in the system boundaries. The activities related to the fertilizer application, as well as the post-application impacts on the field soil have been excluded from all studied systems. Furthermore, the CO_2_ emissions generated for the treatment of the human waste are considered biogenic and, thus, have not been included in the system boundaries of the sanitation systems.

**Table 1:** Input material and energy flow data for the LCA study per functional unit.^a^

Process	NMT	PFT	UDDT
**Raw material extraction**			

Faeces (kg)	2.00E+00^1^	2.00E+00^1^	2.00E+00^1^
N (kg)	1.10E-02^2^	1.10E-02^2^	1.10E-02^2^
P (kg)	5.48E-03^2^	5.48E-03^2^	5.48E-03^2^
K (kg)	1.10E-02^2^	1.10E-02^2^	1.10E-02^2^
Urine (kg)	1.42E+01^3^	1.42E+01^3^	1.42E+01^3^
N (kg)	8.22E-02^2^	8.22E-02^2^	8.22E-02^2^
P (kg)	8.22E-03^2^	8.22E-03^2^	8.22E-03^2^
K (kg)	3.29E-02^2^	3.29E-02^2^	3.29E-02^2^
Polystyrene - toilet seat (kg)	9.39E-04^4^	3.65E-04^4^	3.65E-04^4^
Polystyrene - cistern (kg)	1.64E-03^4^	-	-
Glass fibre - membrane (kg)	1.45E-05^5^	-	-
Alloy steel - combustor (kg)	3.32E-04^5^	-	-
Alloy steel – screw (kg)	4.20E-04^5^	-	


**Manufacture**			

Injection moulding (kg)	3.33E-03	3.65E-04	3.65E-04
Sanitary ceramics - toilet bowl (kg)	8.45E-03^4^	3.29E-03^4^	3.29E-03^4^
Transportation (t-km)	1.11E-02	3.43E-03	3.43E-03

**Operation & Maintenance**			
Glass fibre (kg)	6.17E-04	-	-
Water (kg)	-	1.50E+02	-
Ash (kg)	8.00E-02^1^	-	-
P (kg)	1.10E-02^6^	-	-
K (kg)	1.21E-02^6^	-	-
NOx-emissions (kg)	1.10E-02^1^		


**Waste Management**			

Transportation (t-km)	-	3.82E+00	5.00E-02
Electricity (kWh)		6.80E-02^7^	6.60E-03^10^
Diesel (kg)	-	-	1.11E-02^10^
Lime (kg)	-	2.00E-03^8^	-
Polymer (kg)	-	2.14E-05^8^	-
Iron chloride (FeCl_3_) (kg)	-	1.42E-05^8^	-
NH_3_-emissions (kg)	-	-	1.42E-03^11^
CH_4_-emissions(kg)	-	-	8.00E-03^12^
N_2_O-emissions (kg)	-	7.32E-04^9^	4.80E-04^13^


**Product**			

Transportation	2.72E-03	3.60E-04^14^	4.80E-01^15^
N-Fertilizer (kg)	-	1.85E-02^17^, ^18^	7.88E-02^16^, ^19^
P-Fertilizer (kg)	1.04E-02^16^	8.63E-03^17^, ^18^	1.33E-02^16^, ^20^
K-Fertilizer (kg)	1.21E-02^16^	-	4.30E-02^16^, ^20^
Electricity (kWh)	4.62E-02^1^	-	-
Treated wastewater (kg)	9.56E+00^1^	1.50E+02	-

1 (Hanak et al., 2016).

2 (Rose et al., 2015) for a densityUrine=1.002 g/cm^3^.

3 (Jönsson and Vinnerås, 2004).

4 (Genty et al., 2014)

5 Estimations from NMT-Project.

6 P and K content estimated as a percentage of 13.7% and 15.1% of total ash, respectively (Onabanjo et al., 2017).

7 Energy consumption of 590.69 kwh/Ml for an activated sludge treatment plant (Scheepers and van Der Merwe-Botha, 2012).

8 Data adopted from a WWTP with capacity of 50,000–100,000 IE treating nitrogen and/or phosphorus for a total influent volume of 1.15E-01 m^3^ [mass_faecal sludge_ = 166.21 kg; density_faecal sludge_ = 1443.1 kg/m^3^(Niwagaba et al., 2014)] (Lassaux et al., 2007).

9 Eq. 6.8 (Doorn et al., 2006).

10 (van Haaren, 2009).

11 Estimated as 13% of the total input N content (Hao and Benke, 2008).

12 Estimated based on the Eq. (4.1), for an EF = 4 g CH4/kg waste treated [composting; on wet weight basis; Mi = amount of faeces expressed in Gg and *R* = 0 (without gas recovery)] (Pipatti et al., 2006).

13 Estimated based on the eq. (4.2), for an EF = 0.24 g N_2_O/kg waste treated [composting on wet weight basis and M_i_ = amount of faeces expressed in Gg] (Pipatti et al., 2006).

14 Estimated for a sewage sludge of 2.76E-02 kg [sewage sludge production rate of 0.24 kg·m^3^ of treated wastewater](Gurjar and Tyagi, 2017).

15 Estimated for a compost weight of 50% of the faecal matter (Miller and Jones, 1995).

16 N, P and K fertilizers for ash and compost application estimated as a percentage of 45%, 95% and 100% of the applied nutrient content, respectively [average value of mid-term period values] (Lazcano et al., 2014).

17 N and P content in sewage sludge estimated as a percentage of 40% and 90% of the influent nutrient content (From wastewater to eco-friendly fertilizer, 2016).

18 N and P fertilizers estimated as a percentage of 50% and 70% of the respective applied nutrient content (Hospido et al., 2008)**.**

19 N fertilizer from urine application estimated as 90% of the urine N content (Karak and Bhattacharyya, 2011).

20 P and K fertilizers from urine application estimated as 100% of the respective urine nutrient content (Kirchmann and Pettersson, 1995).

#### 2.5.1 Inventory analysis

The life cycle inventory analysis has been implemented based on an attributional approach. The input material and energy flows of the studied systems have been extracted from relevant literature and have been normalized against the reference flow, as defined in the Section 2.1.1. More precisely, in the case of the raw material extraction and manufacture stages of the examined sanitation systems, mass and energy input has been amortized against the lifetime of each toilet system. Moreover, considering that background data specifically for the country of South Africa were not available in the Ecoinvent 3.0 dataset (Ecoinvent, 2018) embedded in SimaPro 8.0 software, generic datasets have been employed for the LCA modelling which are presented in Table S1 (Supporting material).

The material and energy input data for the LCA modelling are presented in Table 1. The values for the mass and energy flow exchanges involved in the raw material extraction, manufacture, use and waste treatment/disposal phases of all three toilet systems have been derived from literature. The input data of the faeces and urine streams employed in the use phase have been adopted from research conducted for the NMT (Hanak et al., 2016; Rose et al., 2015) and have been similarly applied to both the UDDT and PFT systems.

#### 2.5.2 Impact assessment

In order to facilitate the combination of QMRA results - expressed in DALYs as it will be discussed below – with the LCA results, the ReCiPe method, which expresses the impact on human health in the same unit (DALYs), has been selected for the environmental assessment of the three examined sanitation technologies. The specific impact category methodology has been also adopted by similar environmental studies (Harder et al., 2014; Heimersson et al., 2014; Kobayashi et al., 2015; Harder et al., 2015). An endpoint approach and a hierarchist perspective have been adopted for the impact characterization (Goedkoop and Huijbregts, 2013). The following endpoint impact categories have been selected for the evaluation: Damage to Human Health, Damage to Resources and Damage to Ecosystems.

#### 2.5.3 Interpretation

The interpretation of the LCA results, presented in a graphical form below, centres around the individual contribution of the unit processes involved in the studied sanitation systems to each endpoint impact category. In order to facilitate the comprehension of the LCA graphs, a specific categorization of the material and energy flows has been employed. More precisely, for every life cycle stage the cumulative impact of the relevant energy and material flows - as presented in Table 1 - has been reflected in the graphs. The only exception to this categorization are the products of each sanitation system whose impact has been individually reflected in the LCA results. The contribution of each individual exchange and unit process on the LCIA profile of all examined sanitation systems is provided in the supporting material in a tabular form (Tables S4, S5 and S6).

The by-products, i.e. compost, sewage sludge, ash and urine, generated from the studied systems serve as substitutes for commercial mineral fertilizers. The environmental impacts being avoided by this substitution-production of mineral fertilizers- have been credited (LCA credits) to the respective examined systems according to the concept of the “avoided burden” (Azapagic and Clift, 1999; Brander and Wylie, 2011). The same approach has also been employed in the case of the treated wastewater and the produced electricity. Based on this consideration, the LCA results presented in the Section 3 have reflected the LCA credits with a negative value in the respective graphs, whereas the environmental burdens – LCA burdens – with a positive value. However, the environmental credits linked to the treated wastewater in the case of the PFT system have not been displayed in the LCA results since they were counteracted automatically in the SimaPro software by the environmental burdens of the tap water use.

A sensitivity analysis has also been implemented in this study as a means of evaluating the quality of the input data (Björklund, 2002). Based on the initial LCA simulations, certain system parameters have been identified as critical for the robustness of the LCA model and, thus, have been evaluated for a ±20% variation in their initial value. More precisely, the selected parameters are: a) the transportation involved in the waste management of the UDDT and PFT systems b) the amount of electricity generated in the NMT systems c) the amount of NO_X_ gases emitted from the NMT system and d) the nutrient content of the human urine and faeces with respect to the fertilizer substitution. The latter parameter has been applied in the case of the NMT system by modifying the respective nutrient content in the ash, as the concentration of the available nutrients has been expressed as a percentage of the total amount of ash rather than the initial feed concentration. In addition to the sensitivity analysis, the pedigree matrix (Table S10 in supporting material) has been employed to quantify the uncertainty of the foreground data (Weidema and Wesnaes, 1996) by means of squared geometric standard deviation (SD^2^). The estimation of the latter has been based on empirical uncertainty factors (Ciroth et al., 2016) and basic uncertainty factors (Frischknecht et al., 2007), which are presented in the supporting material.

### 2.6 Quantitative microbial risk assessment (QMRA)

The QMRA study has appraised the human health risks linked to the exposure to faecal pathogens through different routes, mainly induced by the insufficient operation of the waste and wastewater treatment activities linked to the UDDT and PFT systems. As far as the NMT is concerned, the system is considered capable of eliminating any pathogen existing in human excreta, due to the high temperature achieved by the combustion process, values that reach up to 600 °C. In support of this argument, taking into account the temperature range – from 4 °C to 30 °C – related to the persistence of certain pathogens in sewage (Oakley et al., 2017), application of an approximate 20 times higher temperature can ensure the attainment of such outcome. For that reason, the NMT has not been included in the QMRA study, as 100% removal of pathogen has been assumed. Additionally, exposure to pathogens during the use phase of the examined toilet systems has been excluded from the scope of the given environmental study as it has been assumed to be the same for all systems and, in turn, pose the same risks. Moreover, the cross-contamination of the human urine has been omitted, as storage under specific conditions can render urine sanitized and, in turn, safe for agricultural use (Gaulke et al., 2010; Makaya et al., 2014; Winblad and Simpson-Hébert, 2004). Furthermore, the contact with the faecal matter during the defecation process has also been excluded for all examined systems.

The burden of disease posed by pathogen exposure has been estimated in this QMRA study by conducting Monte Carlo simulations (10,000 iterations) for certain stochastic input parameters that are presented in the section below.

#### 2.6.1 Hazard identification

The prevalence of human enteric pathogens in the surface water specifically for sub-Saharan countries, including South Africa, has been extensively reported in literature and has been identified as one of the leading causes of diarrhoeal disease (Chattaway et al., 2016; Langendorf et al., 2015; Ouédraogo et al., 2016). In the context of this QMRA study, five pathogens responsible for diarrhoea infection have been selected: *Enterotoxigenic E. Coli, Shigella spp., Cryptosporidium spp., Norovirus and Rotavirus* (Julian, 2016). Considering that coherent data on the concentration of these pathogens in adult faecal matter were not available, relevant faecal shedding rates from cases of diarrhoea disease in children have been adopted (Julian, 2016).

The concentration of the waterborne pathogens in the local river, where the WWTP of this study discharges the effluent, has been estimated based on Eq. (2) of the given reference (Keller et al., 2014), where the median value (DF = 37.00) has been employed as a dilution factor, and the influent of the WWTP has been assumed to contain the same amount of pathogens as present in the faeces after defecation, without the consideration of any growth or inactivation of the pathogen population prior to wastewater treatment, as shown in Table 2. An overall pathogen removal efficiency of 91% has been assumed, as adopted from the respective removal of coliphages found in literature (Momba et al., 2009).

**Table 2:** Pathogen concentration in faeces and river water expressed in [# of pathogens/mg faeces or ml water].

Pathogen	Faecal concentration	Water concentration (River)
Enterotoxigenic *E. coli*	1.00E+04 - 1.00+05	3.36E+02 - 3.36E+03
*Shigella spp*.	1.00E+01 - 1.00E+02	3.36E–01 - 3.36E+00
*Cryptosporidium spp.*	1.00E+00 - 1.00E+04	3.36E–02 - 3.36E+02
Norovirus	1.00E+04 - 1.00E+05	3.36E+02 - 3.36E+03
Rotavirus	1.00E+02 - 1.00E+07	3.36E+00 - 3.36E+05

In the case of the composting plants, many of the research studies on pathogen removal have reported almost a 100% removal of pathogens by the end of the composting process (Jones and Martin, 2003; Shanahan et al., 2010; Sobsey et al., 2003). There are only few references on the survival of Helminth eggs in the mature compost (Burton and Turner, 2003; Havelaar et al., 1985). However, this hazard has not been yet incorporated into the QMRA in such comprehensive manner. On these grounds, a complete pathogen removal in the windrow composting considered in this case study has been assumed. The concentration of pathogens in faeces prior to treatment follows the same assumption as applied to the WWTP.

#### 2.6.2 Dose exposure

The exposure to the pathogens examined in this QMRA study takes place through three different pathways for the case of the WWTP plant: 1) Ingestion of wastewater during handling the feacal sludge (Route 1) 2) Unintentional ingestion of water from recreational and household activities- such as dish and clothes washing and etc. - in the local river (Route 2) and 3) Intentional consumption of water –as a drinking source– from the local river (Route 3). The dose of each pathogen (Dose_pathogen_) ingested by the receptor through the consumed water is given by Eq. 1:

(1) $${\mathrm{Dose}}_{\mathrm{pathogen}}=C_{\mathrm{pathogen}\left(\mathrm{water}\right)}\times V_{\mathrm{water}}$$

where C_pathogen(water)_ is the pathogen concentration in water and V_water_ is the volume of consumed water.

In this study, the volume of the consumed water (*V*_*water*_) has been selected from literature (Steyn et al., 2004), specifically reflecting the prevailing living conditions in South Africa. For Route 1, 5 ml of wastewater has been assumed to be ingested, whereas for Routes 2 and 3 the ingested volume has been set in the range of 10 to 100 ml and 630 to 952 ml (Labite et al., 2010), respectively, covering the majority of the possible recreational activities- and all age groups except for the group of infants.

In the case of the composting plant, the following exposure route has been considered: ingestion of faeces during the handling of the faecal matter (Route 4). The ingested dose is estimated similarly to the above equation, with the volume of water being replaced by the intake of ingested faeces which ranges from 8 to 134 mg (adults, raw material) (Schönning et al., 2007).

#### 2.6.3 Dose response

The probability of infection from ingesting a dose of each pathogen in a single event of exposure to one of the aforementioned routes has been modelled by functions well-established in QMRA analyses (Chaudhry et al., 2017; Haas et al., 1999). For the Enterotoxigenic *E. coli*, *Shigella spp*. and Rotavirus, the Beta-Poisson function has been employed, as defined by the Eq. 2. In the case of *Cryptosporidium spp.* the one-hit exponential dose-response function has been used Eq. 3, whereas for the Norovirus the 1F1 hypergeomeotric function has been applied (Eq. 3) (Van Abel et al., 2017).

(2) PINFECTION=1−1+DoseN50×21α−1−α

(3) $$P_{\mathrm{INFECTION}}=1-\exp\left(-r\cdot \mathrm{Dose}\right)$$

$$P_{\mathrm{INFECTION}}=1{-}_{1}F_{1}\left(\alpha ,\alpha +\beta ,-\mathrm{Dose}\right)$$

where

(4) β=N50212−1

where α, β, N50 are specific dose-response parameters.

The annual risk of infection is given by Eq. 5, where *f*_*exp*_ is the number of exposures to a single pathogen in a year.

(5) $$P_{\mathrm{ANNUAL}-\mathrm{INFECTION}}=1-{\left(1-P_{\mathrm{INFECTION}}\right)}^{f_{\exp}}$$

In the context of this QMRA study, the frequency of exposure has been assumed to be 1 event for all examined routes so as to reflect the minimum possible risk related to pathogen exposure that can occur within the defined LCA system boundaries. Based on this assumption, the annual risk of infection becomes equal to the risk of infection to a single exposure event**.**

#### 2.6.4 Risk characterization

Risk characterization entails the integration of the information provided from the aforementioned hazard characterization, dose-exposure and dose-response steps, to quantify the effects on human health after exposure to pathogens in disability-adjusted life years (DALYs). The equation employed for the estimation of the total burden of disease in DALYs per person per year is given by Eq. 6 (Katukiza et al., 2014):

(6) $$\mathrm{DALY}s=\sum P\left(\mathrm{ill}/\inf\right)\times P_{\mathrm{ANNUAL}-\mathrm{INFECTION}}\times {\mathrm{BOD}}_{\mathrm{PATHOGEN}}$$

where *P(ill|inf)* denotes the probability of illness after infection, *P*_*ANNUAL-INFECTION*_ the annual probability of infection and *BOD*_*PATHOGEN*_ the burden of diarrheal disease resulting from the respective pathogen. Similarly to the Eq. 5, the employment of the risk of infection for a single event in Eq. 6 results in the total burden of disease per person for a single exposure event. The values of the dose-response parameters have been derived from literature and are presented in Tables S2 and S3 (supporting material).

In order to aggregate the impacts generated from the LCA study with those from the QMRA, the unit of the latter should be aligned with the unit of the LCA results. A common practice, adopted by the majority of the reported literature (Harder et al., 2015, Harder et al., 2014; Kobayashi et al., 2015), to address this issue is the expression of the functional unit of the LCA study and the QMRA results on an annual basis. However, adopting a similar approach in this research work would conflict with the principal scope of this LCA study, as strongly underpinned by the functional unit. To elaborate, the functional unit of this LCA study has been strictly defined as the provision of a specific treatment - imposed by each examined sanitation technology - of human waste produced on a daily basis. Based on this selection, the duration of those specific treatment processes has been set outside of the scope of this study since each one operates under a different time framework. In addition to that, considering that the estimation of the material and energy requirements, as well as the emissions of the relevant treatment processes is based solely on the total amount of handled waste rather than any time related basis, amortising these data over a day or a year seems to be an arbitrary and misleading approach to the representation and assessment of the respective environmental impacts. For that reason, in order to enable aggregation of the LCA and QMRA results in this case, the assumption of a single exposure event taking place in the context of the defined functional unit has been made. The total burden of disease, as estimated by Eq. 6, has been multiplied by the number of household members exposed to routes described above. For Route 1 and 4 two household occupants, serving the role of the cesspit emptiers, have been assumed to be exposed, whereas for Routes 2 and 3 all ten household members have been considered.

## 3 Results

### 3.1 LCA results

#### 3.1.1 Human health impact category

**Fig. 4:**
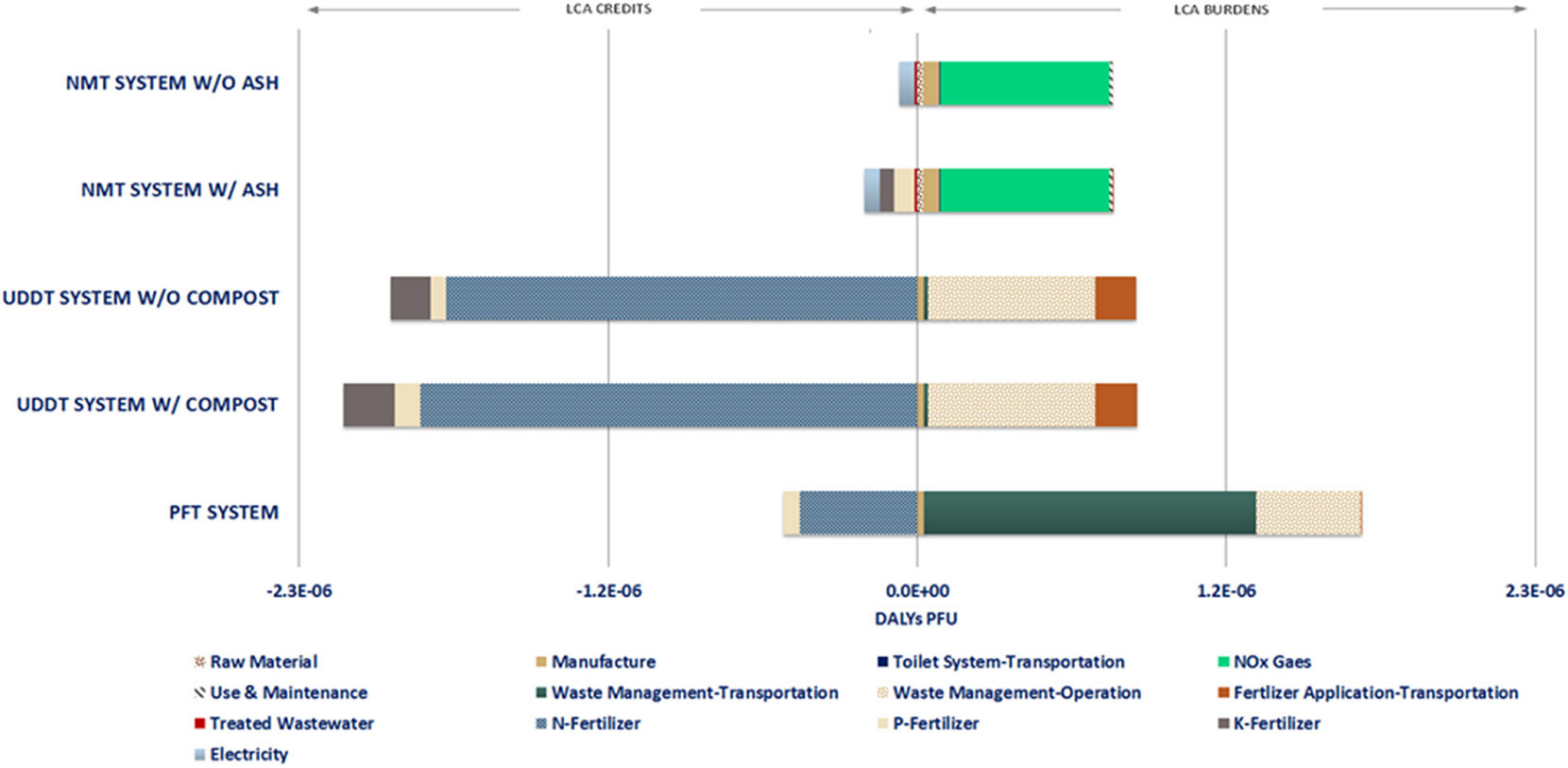
Contribution of life-cycle phases to human health impact category expressed in DALYs per functional unit (PFU).

The absolute impacts of the life cycle stages for each sanitation system on the human health impact category are presented in Fig. 4. As it can be deduced from the distribution of the environmental burdens and credits among the different life cycle phases, the use phase dominates the profile of the NMT system - without the consideration of the ash- by a percentage of 79%, which is mainly attributed to the NO_X_ gases generated during the faeces gasification (Table S4 in supporting material). The second most important contributory factor is the manufacture phase with a share of 8%, whereas the transportation of the toilet system and the maintenance have a minimal impact. Overall, the impact of the credits –negative contribution- linked to the product of the NMT system constitutes 8% of the absolute value, with the greatest contribution deriving from the electricity production. The application of the generated ash waste stream as fertilizer improves the absolute value of the given impact category by 19%. The major contribution to the relevant environmental credits derives from the P-fertilizer with a share of 8%.

In the case of the PFT system, the impact category profile is highly dictated by the impact of the waste management and, more specifically, by that of the transportation which accounts for 57% of the absolute value. The operation of the WWTP plant is responsible for 18% of the given LCIA profile, offsetting completely the credits associated with the use of the sewage sludge as fertilizer and resulting into an overall positive absolute value for the specific category. On the other hand, the UDDT system, in both design scenarios considering the inclusion and exclusion of ash as fertilizer, exhibits an overall negative value for the specific impact category, mainly due to the predominance of the product benefits over the waste management phase accounting for approximately 71%. The incorporation of the compost as fertilizer in the context of the UDDT system has increased the cumulative impact of the fertilizer value of its products by 8% as compared to the initial system, resulting in a 15% decrease in the absolute value of the human health impact category. Nevertheless, with respect to the burdens linked to the waste management, it is apparent that the effect of the operation of the composting plant is quite significant and quantitatively similar to that of the use phase in the NMT system.

In general, the influence of the raw material extraction and manufacture phases on the human health category seems to be relatively minimal for almost all sanitation systems, ranging from 0%–3% and 1–8%, respectively. At a comparative level, the UDDT system exhibits the lower absolute value for the given impact category, whereas the NMT system comes in the second place with an impact category value lower compared to the PFT by 43% and 54%, respectively for the scenario without and with the inclusion of ash, and higher as compared to both UDDT system scenarios averagely by 154% and 144%.

#### 3.1.2 Resources impact category

**Fig. 5:**
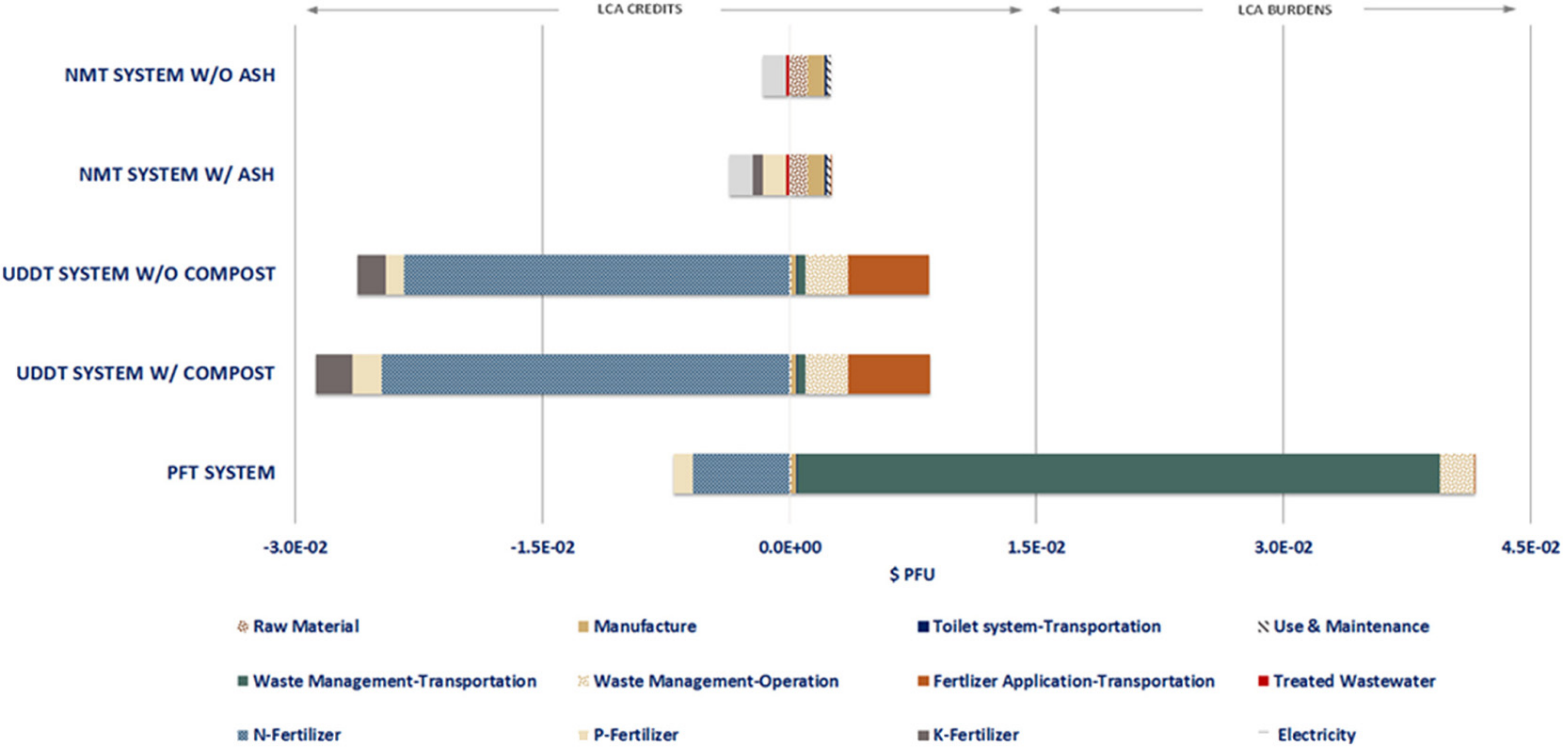
Contribution of life-cycle phases to resources impact category expressed in $ per functional unit (PFU).

The contribution of the life cycle phases of all examined sanitation systems to the damage to resources impact category is depicted in Fig. 5. As far as the NMT system is concerned, the use phase, dictated primarily by the NOx gases, demonstrates no effect on the given impact category while the other phases retain the same impact profile. This is mainly attributed to the absence of effect of the NOx emissions on the midpoint impact indicators involved in the resources impact category (Goedkoop and Huijbregts, 2013; Rijksinstituut voor Volksgezondheid en Milieu (RIVM), 2018). By excluding the use of the ash product, this results in the system exhibiting a marginally positive value with a cumulative share of the product credits accounting for 39% of the total impacts. However, the inclusion of ash as potential fertilizer increases the contribution of the environmental credits to 59%, rendering a negative absolute value of the specific impact category.

In terms of the conventional sanitation technologies, although the contribution of the waste management operation seems to be reduced for both PFT and UDDT systems, the impact of the transportation involved in the waste management and fertilizer application is intensified. Comparatively to the contribution demonstrated in the human health category, the transportation of the fertilizers to the selected field, in the case of the UDDT system, shows an increase from 5% to 13%, for the scenario including only the urine, and from 6% to 14% for the scenario including both compost and urine products. As far as the PFT system is concerned, the absolute value of the impact category continues to be positive and dominated by the effect of the transportation related to waste management by 80% as compared to the respective share of 18% exhibited in the human health category.

Regarding the relative performance of the systems against the resources impact category, a similar profile to that of the human health impact category is followed. The NMT system considering the ash utilization demonstrates a value of the resources impact category higher by 95% than the corresponding values of both UDDT system scenarios, while the exclusion of such consideration results in a corresponding value higher by 105%. With respect to the PFT system, the NMT system shows a lower value for its respective design scenarios by 103% and 97%.

#### 3.1.3 Ecosystems impact category

**Fig. 6:**
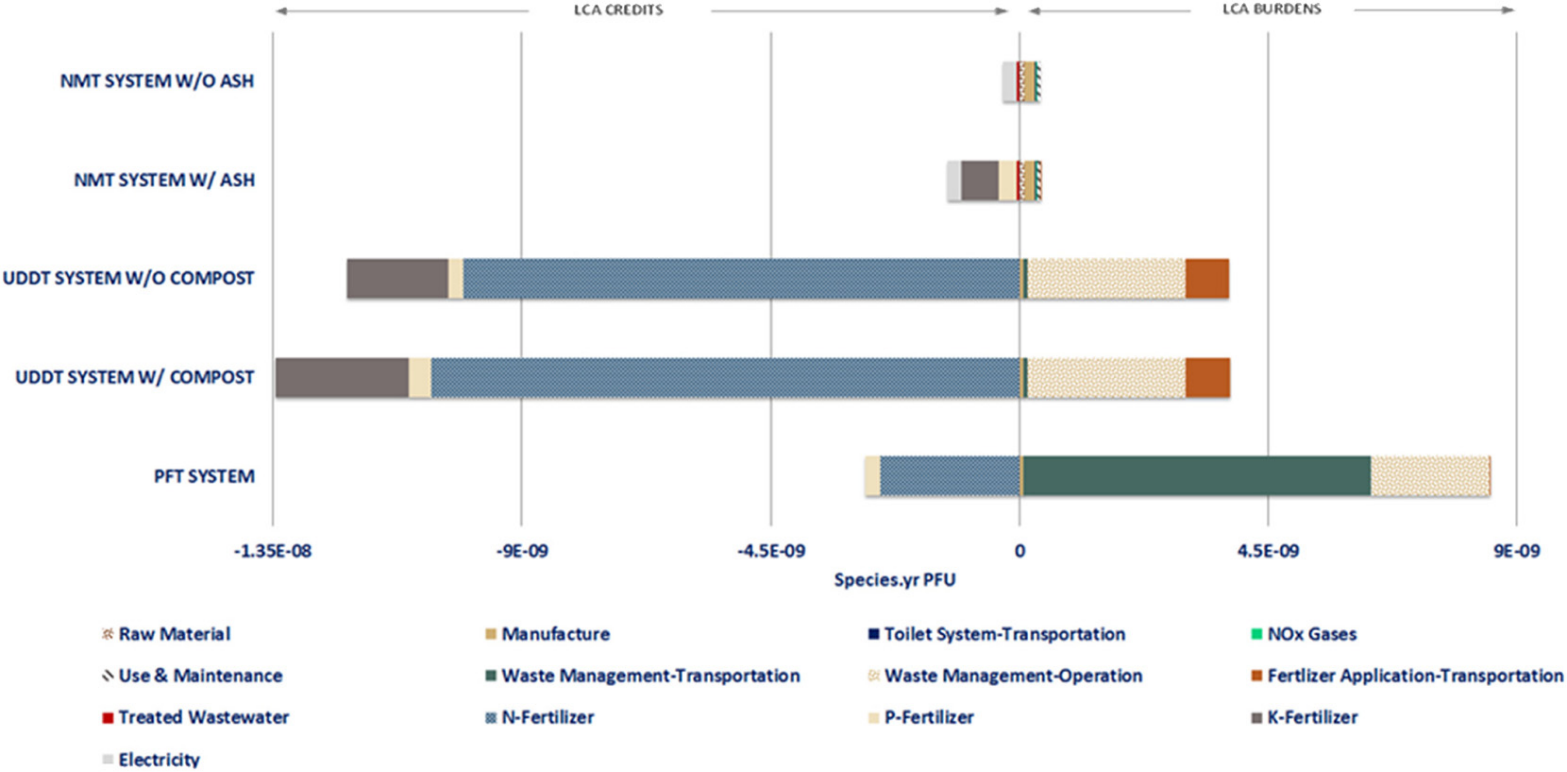
Contribution of life-cycle phases to ecosystems impact category expressed in Species.yr per functional unit (PFU).

In the case of the damage to ecosystems impact category, as depicted in Fig. 6, the NMT system demonstrates an overall positive and negative value for the scenarios where ash is excluded and included, respectively. It is worth mentioning that, though the distribution of the environmental impacts over the major LCA phases follows the same pattern as to that of the two aforementioned impact categories, a significant increase in the impact of the K-fertilizer is observed in the examined sanitation systems. More specifically, the relative contribution has been increased to the range of 12%–40% as compared to the respective range of 5%–6% and 5%–10% exhibited in the damage to human health and resources impact categories.

At a comparative level and for the given impact category, the NMT system seems to environmentally underperform as compared to the UDDT system with a relevant impact category value lower by 101% and 90% for the NMT system scenarios discussed above. Nonetheless, when compared with the PFT, the NMT system depicts a better environmental performance by 99% and 116% for its corresponding design scenarios.

### 3.2 Sensitivity analysis

**Fig. 7:**
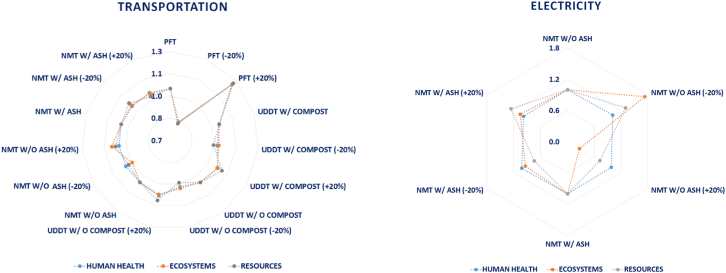
Impact of a) transportation and b) electricity on the LCIA profiles of the sanitation systems (sensitivity scenarios normalized against initial conditions of each sanitation system).

The impact of data uncertainty on the LCIA profile of the examined sanitation systems has been evaluated with respect to certain process related factors whose contribution has been identified as critical based on the discussion provided in Section 3.1. To elaborate, the impact of transportation on the three impact categories has been investigated for the NMT, PFT and UDDT systems. As it can be deduced from Fig. 7, by changing the transportation input by ±20%, no particular change can be observed in the LCIA profile of the NMT system without the consideration of the ash product for the impact categories of damage to human health and ecosystems. A minor change can be observed in the damage to resources impact category which accounts for ±3%. For the rest of the systems, except for the PFT, the variation in their LCIA profile is also relatively small, ranging from ±2% to ±6%. Nonetheless, in the case of the PFT system considerable deviation in the respective profile can be detected in the range of ±21%–±22%. This clearly indicates the importance of the mass handled and the distance travelled in the context of the PFT system on its overall environmental profile.

**Fig. 8:**
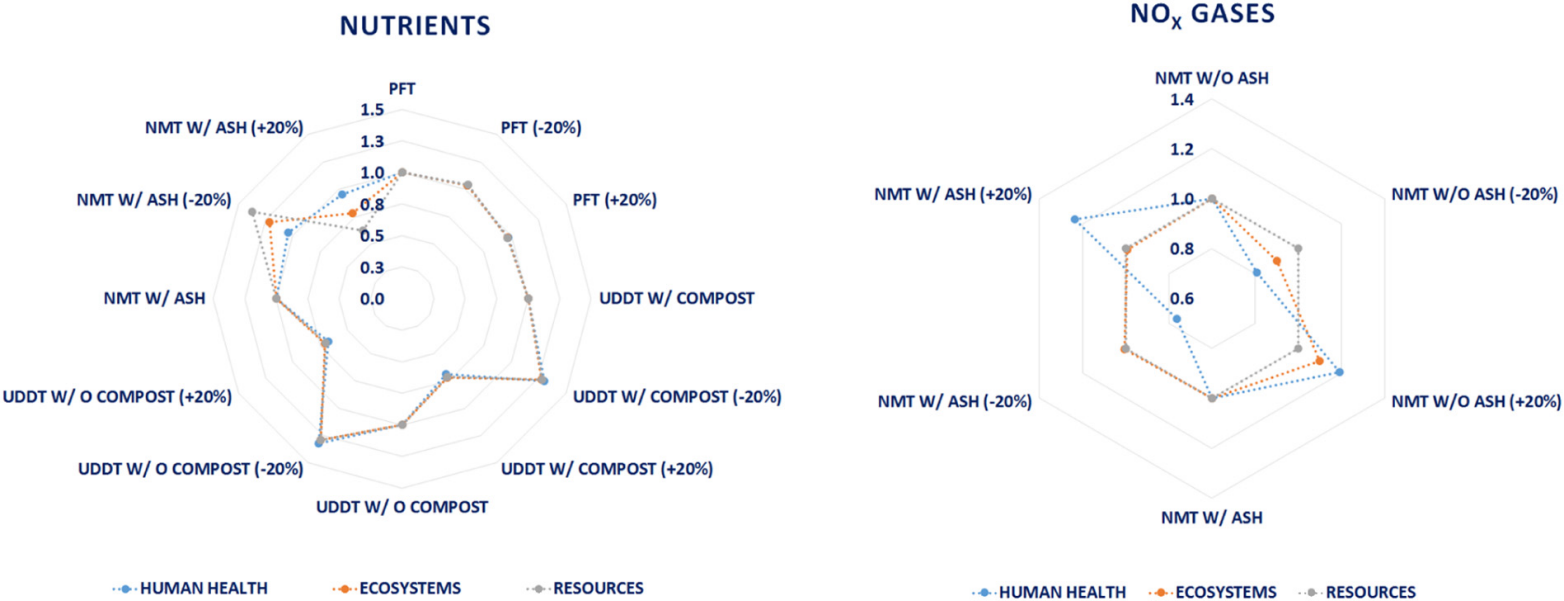
Impact of c) nutrients content and d) NO_X_ gases on the LCIA profiles of the sanitation systems (sensitivity scenarios normalized against initial conditions of each sanitation system).

The same input data variation has been applied to the amount of electricity generated by the NMT system, as illustrated in the Fig. 8, and a different profile has been detected in the two design scenarios based on the utilization of the ash as fertilizer. To elaborate, in the case of no use of the ash product, the human health impact category seems not to be affected significantly, only by 2%. However, the remaining two impact categories, damage to ecosystems and damage to resources, demonstrate great vulnerability at the change of the electricity generated, accounting for ±73% and ±30%, respectively. For the scenario where ash is deployed as fertilizer, the same percentage variation of ±2% is exhibited for the human health category, whereas for the other two categories it is ±6% and ±26%, respectively.

In the case of the amount of emitted NO_X_ gases, variation in the input data seems to affect only the human health and the ecosystems impact categories. Among the two different system scenarios considered for the NMT, the one which omits the use of ash as fertilizer, presents a corresponding deviation of ±19% and ±10% in the values of the impact categories. On the other hand, the other scenario considering the fertilizer value of the ash exhibits a variation of ±24% and ±1%, respectively.

As far as the variation in the nutrient concentration of the urinal and faecal matter is concerned, the impact on the LCIA profile of all sanitation systems is quite significant, demonstrating the criticality of the particular process parameter in the LCA results. More precisely, for the NMT system utilizing the ash product, its profile shows a small variation for the human health category, ±5%, while for the ecosystems and resources the respective variation is ±22% and ±38%. The profile of the UDDT system appears under the influence of the nutrient content to behave in the same manner for all impact categories, ranging from ±29% to ±32%. On the contrary, the PFT demonstrates great robustness against the fluctuation of the nutrient concentration since no difference is observed in its respective LCIA profile.

### 3.3 QMRA-pathogen risk

In this section, the incorporation of the pathogen risk into the LCA has been considered for the PFT and UDDT –including the compost product as fertilizer- systems, and the updated LCIA profiles have been subsequently compared against that of the NMT -with the inclusion of ash- system. The burden of disease as estimated from the Monte Carlo simulations is presented in Table 3, expressed in DALYs for all pathogens under the selected exposure routes. The total DALYs estimated from the QMRA for the Routes 1 to 3 have been aggregated to a single value under the pathogen risk of the FT system. On the contrary, the UDDT system incorporates the total DALYs generated through Route 4.

**Table 3:** Pathogen risk estimated in DALYs for each examined exposure route from Monte Carlo Simulations (median values).

Pathogen	Route 1	Route 2	Route 3	Route 4
Enterotoxigenic *E. coli*	8.36E-05	5.32E-06	5.26E-05	7.84E-05
*Shigella spp*.	3.62E-05	7.99E-06	2.98E-05	3.53E-05
*Cryptosporidium spp*.	6.10E-04	6.10E-04	6.10E-04	6.10E-04
Norovirus	2.43E-04	1.96E-04	2.32E-04	2.41E-04
Rotavirus	1.35E-04	1.34E-04	1.35E-04	1.35E-04
Total risk (per person)	1.11E-03	9.52E-04	1.06E-03	1.10E-03
Total risk (affected population)	2.22E-03	9.52E-03	1.06E-02	2.20E-03

**Fig. 9:**
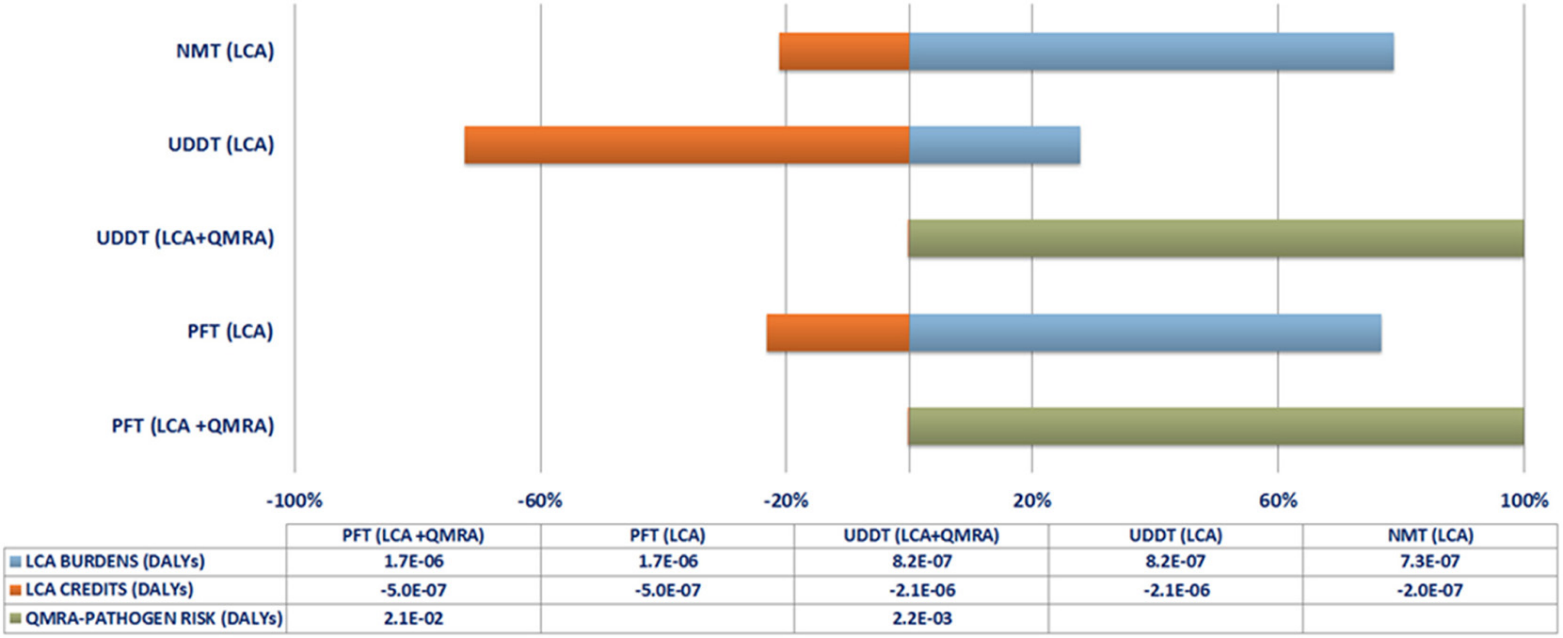
Normalized percentage contribution of environmental credits, burdens and pathogen risk to human health impact category for the NMT (w/ ash), UDDT (w/ compost) and PFT systems (absolute values are presented in the table under the relevant graph).

Fig. 9 presents the percentage contribution of the relevant LCA burdens and credits to the impact category of human health, prior (LCA) and after the incorporation of the pathogen risk (LCA + QMRA). As it can be concluded from the graph, the inclusion of the pathogen risk increases the impact on human health, outweighing practically any benefit associated with the use of urea as fertilizer, as previously discussed for both UDDT and PFT systems. More precisely, the value of the human health impact category for the PFT and UDDT is 4E + 04 and 4E + 03 times higher than that of the NMT system. Nonetheless, the case of the UDDT shows a relatively lower pathogen risk as to the PFT, which is attributed to the lower health risk and number of people exposed to the given route. However, considering that the upper limit for the tolerable disease of burden is set to 10^−6^ DALYs pppy (per person per year) based on the WHO Guidelines (WHO, 2010), important indications are provided with respect to the health challenges posed by the examined sanitation systems.

A sensitivity analysis has been carried out by altering the exposure frequency to five events and the illness to infection ratio by +20% for all pathogens (Tables S8 and S9). In the case of the exposure frequency, the five times increase of the exposure events has resulted in an increase of the total impact of the pathogen risk by 32% and 29% for the PFT and UDDT systems, respectively. Despite the influence of this parameter on the QMRA results, it is important to be mentioned that the selection of the number of events and people exposed to pathogens is primarily subject to the scope of the QMRA study and the considered geographical location. For the reasons mentioned above, the selection of the number of events and people exposed has been dictated in this research work by the functional unit defined in the LCA study. In terms of the illness to infection ratio, the applied change to its value has posed an approximate proportional increase in the impact of the pathogen risk by 22% and 17% for the PFT and UDDT systems, correspondingly. This indicates the importance of the specific parameter in the overall uncertainty of the QMRA results (Itoh, 2013).

Although the human health impact category constitutes an important aspect to consider in an environmental assessment, it is not the only factor determining the environmental performance of a system. It is important to be mentioned that though the given QMRA study enhances the environmental assessment of different sanitation systems by capturing and evaluating holistically the potential human health impacts associated, directly or indirectly, with their use, it incommodes the aggregation of the three impact categories to a single score and, in turn, the overall environmental appraisal and comparison of the studied systems. This is attributed to the lack of normalization and weighting data for the impact of the pathogen risk. For that reason, in order to provide a complete environmental study, the evaluation of the sanitation systems against the other environmental impact categories-apart from human health- imposed by the selected LCA methodology has also been presented in Section 3.1.

## 4 Discussion

Although LCA is considered as the most comprehensive approach to the assessment of environmental impacts, it is also associated with certain limitations (Björklund, 2002). Reliability of LCA is often subject to debate due to the lack of accurate and representative data. In the context of this study, the sensitivity analysis along with the pedigree matrix, provided in Tables S7, S8, S9 and S11 (supporting material) have highlighted the importance of specific input data on the overall credibility of the LCA results. More precisely, in the case of the NMT system the low quality of input data for the NOx emissions and the electricity, deriving primarily from the process simulations (Hanak et al., 2016), in combination with their relatively high contribution to the three examined impact categories, renders the specific flows critical for the reliability of the LCA results. This can be potentially eliminated by providing more accurate data based on laboratory experiments or on-site testing of the NMT system. The same concept applies to the fertilizer products whose contribution to the LCIA profile of all sanitation systems is quite significant. However, in that case a systematic approach is required with respect to the collection of representative data on the nutrient mineralization factors since the data employed for the nutrient content in human excreta has been largely representative (Rose et al., 2015; Jönsson and Vinnerås, 2004).

In terms of the input raw material, the quality of the data provided in the LCA models is also low, yet its contribution is not so significant as compared to the aforementioned material and energy flows. This renders the data uncertainty of the particular parameter of lower importance since its environmental impact is amortized over the lifetime of the respective toilet system. The transportation, though it affects considerably the LCIA profile of the conventional sanitation technologies, constitutes an inherently uncertain model parameter, fact which is reflected by the allocated basic uncertainty factor in the pedigree matrix (Table S11). The quality of the given input data is subject to the geographical application context of the LCA study**.** In this research study, transport distances have been estimated by considering the Alice city as reference point, so as to ensure consistency of the data used in both LCA and QMRA analyses.

Despite the limitations reported above, the given LCA study has provided important insights into the relative performance of the selected sanitation systems, with emphasis being placed on the individual contribution of the unit processes involved in their system boundaries. The LCIA profile of the NMT system has been dictated by the NOx emissions and the credits associated with the electricity generation and the ash utilization as fertilizer. These facts highlight the areas of the NMT design that can be potentially improve by incorporating additional design considerations. More precisely, the reduction of the NOx emissions could be possibly attained by employing a special micro-membrane for the treatment of the flue gas. In terms of the generated electricity, minimizing heat losses is of high importance and it can be achieved with the optimization of the feeding mechanism of the combustor and the selection of proper insulating materials that will not compromise the overall reliability of the system. Considering the environmental benefits linked to the use of ash as a mineral fertilizer substitute, a proper human interface collection system should be designed to minimize product loss.

From a technology uptake viewpoint, the environmental performance of a sanitation technology constitutes an important decision factor, but not the determining one. Uptake of a specific sanitation technology is more dependent on economic, location, infrastructure, and user behaviour and aspirational factors. However, Goal 6 of the Sustainable Development Goals (United Nations, 2017) requires governments to ensure availability and sustainable management of water and sanitation for all and in this case the term “sustainability” encompasses environmental protection. As a result, the environmental performance of sanitation systems and their embedded technologies will come under increasing scrutiny in the future. The work described in this paper will not only inform NMT technology development to enhance environmental performance but also form a valuable basis for future sanitation system comparisons. Based on the aforementioned, a holistic approach needs to be adopted for the further development of the NMT system to become a feasible and sustainable sanitation solution.

## 5 Conclusions

Due to the limited water resources and poor municipal infrastructure, a sustainable sanitation system is in urgent need in developing countries, including South Africa. In this study, the self-sustained waterless toilet system, Nano Membrane Toilet (NMT) has been environmentally assessed parallel to conventional sanitation systems, the Pour Flush Toilet (PFT) and the Urine Diverting Dry Toilet (UDDT) in the context of South Africa. In addition to the traditional LCA, a QMRA study has been conducted with the view to encompassing the health risks associated with the existing sanitation practices. This research work has employed a comparative approach to mainly facilitate the identification of the critical aspects of the environmental profile of the NMT system, so that improvements can be made in the early stage of its development. The most important findings from the environmental assessment are summarized as follows:

• At an endpoint level, the human health impact category for the NMT system -without the inclusion of ash as fertilizer- is primarily dictated by the use phase, whereas for the ecosystems impact category the major contributor is the manufacture phase. The impact of the raw material extraction has proven to be minor as compared to the other life cycle stages, showing that the material selection for the NMT system will not significantly affect its environmental profile. For the scenario of incorporating the benefits of using ash as fertilizer, the NMT demonstrates a similar profile for the human heath impact category as to the aforementioned NMT system, whereas for the rest of the categories the dominating factor of its profile is the credits from the fertilizer substitution.
• In the case of the UDDT system, the credits from the N-fertilizer and, in second place, the waste management phase – including transportation and operation – are the major contributors to the absolute values of the impact categories. The performance of the PFT system in all impact categories is dominated by the waste management, and more precisely by the transportation, which outweighs significantly the benefits from the disposal of the sewage sludge as fertilizer.
• The human health impact category of the NMT system, with and without the inclusion of the environmental credits related to the generated ash, against that of the UDDT system has been, respectively, 144% and 154% higher, whereas against that of the PFT system it has been 54% and 43% lower. In the case of the resources impact category, the value of the aforementioned NMT systems is 95% and 105% higher than that of the UDDT system. On the contrary, when compared with the PFT system the respective value of the two examined NMT systems is 103% and 97% lower, correspondingly. Lastly, in terms of the ecosystems impact category, the NMT system scenarios, with and without the inclusion of ash as fertilizer, exhibit a higher value by 90% and 101% as compared to the UDDT system and a lower value by 99% and 106% that that of PFT system.
• Based on the sensitivity analysis, the nutrient content and the NOx gases are the most contributory factors of the LCIA profile of the NMT system. For the PFT system, great impact seems to derive from the transportation parameter while for the other sensitivity parameters no alteration of its profile has been observed. In the case of the UDDT, variation in both the transportation and nutrient content affects its profile quite considerably.
• The application of the QMRA has rendered the human health impact category of the PFT and UDDT systems 4E + 04 and 4E + 03 times higher, respectively, than that of the NMT system. The better performance of the NMT system over the conventional sanitation technologies with respect to the human health impact category, is mainly attributed to the in-situ treatment of faecal pathogens.
• In terms of the areas that have been identified as critical to the environmental improvement of the NMT system, the role of incorporating ash as fertilizer has been found to be important, followed by the impact of the electricity and NO_X_ gases generated by the system. In the case of the ash utilization, though the produced amount may seem relative small for considering its transportation to a local field, a larger application of the NMT technology, e.g. in a centralized system, is likely to support such consideration and yield higher environmental benefits.

## Appendix A Supplementary data

Supplementary material Image 1

## References

[bb0005] African Water Facility (2014). Un-Sewered Sanitation Improvements for the Urban-Poor: Overview of the African Water Facility Project Portfolio.

[bb0010] Anand C., Apul D.S. (2011). Economic and environmental analysis of standard, high efficiency, rainwater flushed, and composting toilets. J. Environ. Manag..

[bb0015] Andersson E. (2015). Turning waste into value: using human urine to enrich soils for sustainable food production in Uganda. J. Clean. Prod..

[bb0020] Ayres R.U. (1995). Life cycle analysis: a critique. Resour. Conserv. Recycl..

[bb5010] Azapagic A., Clift R. (1999). Allocation of environmental burdens in multiple-function systems. J. Clean. Prod..

[bb0025] Benetto E., Nguyen D., Lohmann T., Schmitt B., Schosseler P. (2009). Life cycle assessment of ecological sanitation system for small-scale wastewater treatment. Sci. Total Environ..

[bb0030] Björklund A.E. (2002). Survey of approaches to improve reliability in lca. Int. J. Life Cycle Assess..

[bb0040] Brander M., Wylie C. (2011). The use of substitution in attributional life cycle assessment. Greenh. Gas Meas. Manag..

[bb0045] Brikké F., Bredero M. (2003). Linking Technology Choice with Operation and Maintenance in the Context of Community Water Supply and Sanitation.

[bb0050] Burton C.H., Turner C. (2003). Manure Management: Treatment Strategies for Sustainable Agriculture.

[bb0055] Chattaway M.A., Aboderin A.O., Fashae K., Okoro C.K., Opintan J.A., Okeke I.N. (2016). Fluoroquinolone-resistant enteric bacteria in sub-Saharan Africa: clones, implications and research needs. Front. Microbiol..

[bb0060] Chaudhry R.M., Hamilton K.A., Haas C.N., Nelson K.L. (2017). Drivers of microbial risk for direct potable reuse and de facto reuse treatment schemes: the impacts of source water quality and blending. Int. J. Environ. Res. Public Health.

[bb0065] Ciroth A., Muller S., Weidema B., Lesage P. (2016). Empirically based uncertainty factors for the pedigree matrix in ecoinvent. Int. J. Life Cycle Assess..

[bb0070] Cleansing Service Group Ltd (2013). Septic Tank and Cesspit Emptying Costs. https://www.septictanksandcesspits.com/blog/septic-tank-and-cesspit-emptying-costs/.

[bb0075] David Border Composting Consultancy (2002). Processes and Plant for Waste Composting and Other Aerobic Treatment.

[bb0080] Department of Environment and Conservation NSW (2006). Life Cycle Inventory and Life Cycle Assessment for Windrow Composting Systems.

[bb0085] Devkota J., Schlachter H., Anand C., Phillips R., Apul D. (2013). Development and application of EEAST: a life cycle based model for use of harvested rainwater and composting toilets in buildings. J. Environ. Manag..

[bb0095] Dong S., Li J., Kim M.-H., Park S.-J., Eden J.G., Guest J.S., Nguyen T.H. (2017). Human health trade-offs in the disinfection of wastewater for landscape irrigation: microplasma ozonation vs. chlorination. Environ. Sci. Water Res. Technol..

[bb0100] Doorn M.R.J., Towprayoon S., Manso Vieira S.M., Irving W., Palmer C., Pipatti R., Wang C. (2006). Wastewater Treatment and Discharge.

[bb0105] Dwaf (2012). Sanitation Services – Quality of Sanitation in South Africa Report on the Status of sanitation services in South Africa.

[bb0110] Ecoinvent (2018). The ecoinvent database. https://www.ecoinvent.org/database/database.html.

[bb0115] European Commission (2010). ILCD Handbook - General Guide for Life Cycle Assessment-Detailed Guidance.

[bb0120] Flores A., Rosemarin A., Fenner R. (2009). Evaluating the sustainability of an innovative dry sanitation (Ecosan) system in China as compared to a conventional waterborne sanitation system. Proc. Water Environ. Fed..

[bb0125] Friedrich E., Pillay S., Buckley C.A. (2009). Carbon footprint analysis for increasing water supply and sanitation in South Africa: a case study. J. Clean. Prod..

[bb0130] Frischknecht R., Jungbluth N., Althaus H., Doka G., Dones R., Heck T., Hellweg S., Hischier R., Nemecek T., Rebitzer G., Spielmann M., Wernet G. (2007). Overview and methodology. Ecoinvent Cent.

[bb0135] From wastewater to eco-friendly fertilizer (2016). Filtr.

[bb0140] Fuhrimann S., Winkler M.S., Stalder M., Niwagaba C.B., Babu M., Kabatereine N.B., Halage A.A., Utzinger J., Cissé G., Nauta M. (2016). Disease burden due to gastrointestinal pathogens in a wastewater system in Kampala, Uganda. Microb. Risk Anal..

[bb0145] Gandy S. (2011). Developing an Evidence Base on Toilets and Urinals.

[bb0150] Gao H., Zhou C., Li F., Han B., Li X. (2015). Economic and environmental analysis of five Chinese rural toilet technologies based on the economic input-output life cycle assessment. J. Clean. Prod..

[bb0155] Gaulke L.S., Weiyang X., Scanlon A., Henck A., Hinckley T. (2010). Evaluation criteria for implementation of a sustainable sanitation and wastewater treatment system at Jiuzhaigou National Park, Sichuan Province. Chin. Environ. Manag..

[bb0160] Genty A., Kowalska M., Wolf O. (2014). Developing an evidence base on flushing toilets and urinals. Preliminary Report.

[bb0165] Goedkoop M., Huijbregts M. (2013). ReCiPe 2008 Characterisation.

[bb0170] Graham J.P., Polizzotto M.L. (2013). Pit latrines and their impacts on groundwater quality: a systematic review. Environ. Health Perspect..

[bb0175] Gurjar B.R., Tyagi V.K. (2017). Sludge Management.

[bb0180] Haas C.N., Rose J.B., Gerba C.P. (1999). Quantitative Microbial Risk Assessment.

[bb0185] Hanak D.P., Kolios A.J., Onabanjo T., Wagland S.T., Patchigolla K., Fidalgo B., Manovic V., McAdam E., Parker A., Williams L., Tyrrel S., Cartmell E. (2016). Conceptual energy and water recovery system for self-sustained nano membrane toilet. Energy Convers. Manag..

[bb0190] Hao X., Benke M.B. (2008). Nitrogen Transformation and Losses During Composting and Mitigation Strategies.

[bb0200] Harder R., Heimersson S., Svanström M., Peters G.M. (2014). Including Pathogen Risk in Life Cycle Assessment of Wastewater Management. 1. Estimating the Burden of Disease Associated with Pathogens. Environ. Sci. Technol..

[bb0210] Harder R., Schoen M.E., Peters G.M. (2015). Including pathogen risk in life cycle assessment of wastewater management. Implications for selecting the functional unit. Environ. Sci. Technol..

[bb0215] Harder R., Peters G.M., Molander S., Ashbolt N.J., Svanström M. (2016). Including pathogen risk in life cycle assessment: the effect of modelling choices in the context of sewage sludge management. Int. J. Life Cycle Assess..

[bb0220] Hatfield J.L., Stewart B.A. (1997). Animal Waste Utilization: Effective Use of Manure as a Soil Resource.

[bb0230] Havelaar A.H., L'Hermite P., Strauch D. (1985). Inactivation of Microorganisms in Sewage Sludge by Stabilization Processes.

[bb0205] Heimersson S., Harder R., Gregory M., Peters M.S. (2014). Including Pathogen Risk in Life Cycle Assessment of Wastewater Management. 2. Quantitative Comparison of Pathogen Risk to Other Impacts on Human Health. Environ. Sci. Technol..

[bb0235] Hospido A., Moreira M.T., Feijoo G. (2008). A comparison of municipal wastewater treatment plants for big centres of population in Galicia (Spain). Int. J. Life Cycle Assess..

[bb0240] Huuhtanen S., Laukkanen A. (2009). A Guide to Sanitation and Hygiene in Developing Countries. Updated Version 2.

[bb0245] Itoh S. (2013). Effect of the ratio of illness to infection of campylobacter on the uncertainty of DALYs in drinking water. J. Water Environ. Technol..

[bb0250] Iweriebor B.C., Gaqavu S., Obi L.C., Nwodo U.U., Okoh A.I. (2015). Antibiotic susceptibilities of enterococcus species isolated from hospital and domestic wastewater effluents in Alice, eastern cape province of South Africa. Int. J. Environ. Res. Public Health.

[bb0255] Jones P., Martin M. (2003). A Review of the Literature on the Occurrence and Survival of Pathogens of Animals and Humans in Green Compost, The Waste and Resources Action Programme.

[bb0260] Jönsson H., Vinnerås B. (2004). Adapting the nutrient content of urine and faeces in different countries using FAO and Swedish data. Ecosan–Closing Loop.

[bb0265] Julian T.R. (2016). Environmental transmission of diarrheal pathogens in low and middle income countries. Environ. Sci.: Processes Impacts.

[bb0270] Jurado N., Onabanjo T., Athanasios J., Kolios B.F., Patchigolla K., Wagland S., Parker A., McAdam E., Williams L., Tyrrel S. (2018). Design and commissioning of a multi-configuration pro-totype for thermal conversion of human faeces. Energy Convers. Manag..

[bb0275] Karak T., Bhattacharyya P. (2011). Human urine as a source of alternative natural fertilizer in agriculture: a flight of fancy or an achievable reality. Resour. Conserv. Recycl..

[bb0280] Katukiza A.Y., Ronteltap M., van der Steen P., Foppen J.W.A., Lens P.N.L. (2014). Quantification of microbial risks to human health caused by waterborne viruses and bacteria in an urban slum. J. Appl. Microbiol..

[bb0290] Keller V.D.J., Williams R.J., Lofthouse C., Johnson A.C. (2014). Worldwide estimation of river concentrations of any chemical originating from sewage-treatment plants using dilution factors. Environ. Toxicol. Chem..

[bb0295] Kirchmann H., Pettersson S. (1995). Human urine - chemical-composition and fertilizer use efficiency. Fertil. Res..

[bb0300] Kjellén M., Pensulo C., Nordqvist P., Madeleine F. (2011). Global Review of Sanitation System Trends and Interactions with Menstrual Management Practices.

[bb0305] Kobayashi Y., Peters G.M., Ashbolt N.J., Heimersson S., Svanström M., Khan S.J. (2015). Global and local health burden trade-off through the hybridisation of quantitative microbial risk assessment and life cycle assessment to aid water management. Water Res..

[bb0310] Kohler Co (2014). Environmental Product-Declaration Persuade ® K-4353.

[bb0315] Kolios A., Jiang Y., Somorin T., Sowale A., Anastasopoulou A., Anthony E.J., Fidalgo B., Parker A., McAdam E., Williams L., Collins M., Tyrrel S. (2018). Probabilistic performance assessment of complex energy process systems – the case of a self-sustained sanitation system. Energy Convers. Manag..

[bb0320] Kulak M., Shah N., Sawant N., Unger N., King H. (2017). Technology choices in scaling up sanitation can significantly affect greenhouse gas emissions and the fertiliser gap in India. J. Water Sanit. Hyg. Dev..

[bb0325] Labite H., Lunani I., Van Der Steen P., Vairavamoorthy K., Drechsel P., Lens P. (2010). Quantitative microbial risk analysis to evaluate health effects of interventions in the urban water system of Accra, Ghana. J. Water Health.

[bb0330] Langendorf C., Le Hello S., Moumouni A., Gouali M., Mamaty A.A., Grais R.F., Weill F.X., Page A.L. (2015). Enteric bacterial pathogens in children with diarrhea in Niger: diversity and antimicrobial resistance. PLoS One.

[bb0335] Lassaux S., Renzoni R., Germain A. (2007). LCA case studies life cycle assessment of water from the pumping station to the wastewater treatment plant. Int. J. Life Cycle Assess..

[bb0340] Lazcano C., Martínez-blanco J., Christensen T.H., Muñoz P., Rieradevall J. (2014). Environmental Benefits of Compost use on Land Through LCA – A Review of the Current Gaps.

[bb0345] Makaya J.M., Savadogo A., Somda M.K., Bour J., Barro N., Traoré A.S. (2014). Quality of human urine used as fertilizer: case of an ecological sanitation system in Ouagadougou peri-urban areas-Burkina Faso. J. Environ. Prot..

[bb0355] Miller J.H., Jones N. (1995). Organic and Compost-Based Growing Media for Tree Seedling Nurseries.

[bb0360] Mnkeni P.N.S. (2009). Fertiliser value of human manure from pilot urine-diversion toilets urine-diversion toilets. Water.

[bb0365] Moilwa N. (2007). The Social/Cultural Acceptability of Using Human Excreta (Faeces and Urine) for Food Production in Rural Settlements in South Africa.

[bb0370] Momba M.N.B., Sibewu M., Mandeya A. (2009). Survival of somatic and F-RNA coliphages in treated wastewater effluents and their impact on viral quality of the receiving water bodies in the eastern Cape Province-South Africa. J. Biol. Sci..

[bb0380] Mugivhisa L.L. (2015). An assessment of university students and staff perceptions regarding the use of human urine as a valuable soil nutrient in South Africa. Afr. Health Sci..

[bb0390] Niwagaba C.B., Mbeguere M., Strande L. (2014). Faecal Sludge Quantification, Characterisation and Treatment Objectives, Faecal Sludge Management.

[bb0395] Nyarko, K.B., Buamah, R., Appiah-Effah, F.K.N.N., Owusu-Boakye, E., Afful, K.M., Samwini, N.A., Owusu-Boakye, A., n.d. Latrine Technology Manual (https://www.unicef.org/ghana/resources_11304.html).

[bb0400] Nyenje P.M., Foppen J.W., Uhlenbrook S., Kulabako R., Muwanga A. (2010). Eutrophication and nutrient release in urban areas of sub-Saharan Africa - a review. Sci. Total Environ..

[bb0405] Nyenje P.M., Foppen J.W., Kulabako R., Muwanga A., Uhlenbrook S. (2013). Nutrient pollution in shallow aquifers underlying pit latrines and domestic solid waste dumps in urban slums. J. Environ. Manag..

[bb0410] Oakley S., States U., Von Sperling M. (2017). Part Four. Management of Risk From Excreta and Wastewater Anaerobic Sludge Blanket Reactors.

[bb0415] Onabanjo T., Kolios A.J., Patchigolla K., Wagland S.T., Fidalgo B., Jurado N., Hanak D.P., Manovic V., Parker A., McAdam E., Williams L., Tyrrel S., Cartmell E. (2016). An experimental investigation of the combustion performance of human faeces. Fuel.

[bb0420] Onabanjo T., Patchigolla K., Wagland S.T., Fidalgo B., Kolios A., McAdam E., Parker A., Williams L., Tyrrel S., Cartmell E. (2016). Energy recovery from human faeces via gasification: a thermodynamic equilibrium modelling approach. Energy Convers. Manag..

[bb0425] Onabanjo T., Kolios A.J., Parker A., McAdam E., Williams L., Tyrrel S. (2017). Faecal-wood biomass co-combustion and ash composition analysis. Fuel.

[bb0430] Orner K.D., Mihelcic J.R. (2018). A review of sanitation technologies to achieve multiple sustainable development goals that promote resource recovery. Environ. Sci. Water Res. Technol..

[bb0435] Ouédraogo N., Kaplon J., Bonkoungou I.J.O., Traoré A.S., Pothier P., Barro N., Ambert-Balay K. (2016). Prevalence and genetic diversity of enteric viruses in children with diarrhea in Ouagadougou, Burkina Faso. PLoS One.

[bb0440] Pels J.R., de Nie D.S., Kiel J.H.A. (2005). Utilization of ashes from biomass combustion and gasification. 14th European Biomass Conference & Exhibition.

[bb0445] Petersens E., Beck-friis B. (2012). How to build and maintain a compost for faeces from dry urine diverting toilets. Faecal Composting.

[bb0450] Petterson S.R., Ashbolt N.J. (2016). QMRA and water safety management: review of application in drinking water systems. J. Water Health.

[bb0455] Pickering A.J., Julian T.R., Marks S.J., Mattioli M.C., Boehm A.B., Schwab K.J., Davis J. (2012). Fecal contamination and diarrheal pathogens on surfaces and in soils among Tanzanian households with and without improved sanitation. Environ. Sci. Technol..

[bb0460] Pipatti R., Alves J.W.S., Gao Q., Cabrera C.L., Mareckova K., Oonk H., Scheehle E., Sharma C., Smith A., Svardal P., Yamada M. (2006). Chapter 4 - biological treatment of solid. 2006 IPCC Guidel. Natl. Greenh. Gas Invent.

[bb0470] Remy C., Jekel M. (2007). Ecological Assessment of Selected Alternative Sanitation Concepts Via Life Cycle Assessment.

[bb0475] Remy C., Jekel M. (2008). Sustainable wastewater management: life cycle assessment of conventional and source-separating urban sanitation systems. Water Sci. Technol..

[bb0480] Rieck C., von Müench E., Hoffman H. (2013). Technology Review of Urine-Diverting Dry Toilets (UDDTs).

[bb0485] Rijksinstituut voor Volksgezondheid en Milieu (RIVM) (2018). Life Cycle Assessment (LCA)-Downloads-ReCiPe2016_CFs_20161004. https://www.rivm.nl/Onderwerpen/L/Life_Cycle_Assessment_LCA/Downloads.

[bb0490] Rose C., Parker A., Jefferson B., Cartmell E. (2015). The characterization of feces and urine: a review of the literature to inform advanced treatment technology. Crit. Rev. Environ. Sci. Technol..

[bb0495] Roux P., Boutin C., Risch E., Heduit A., Roux P., Boutin C., Risch E., Life A.H., Lca A. (2011). Life Cycle Environmental Assessment (LCA) of Sanitation Systems Including Sewerage: Case of Vertical Flow Constructed Wetlands Versus Activated Sludge.

[bb5000] Scheepers R., van Der Merwe-Botha M. (2012). Energy optimization considerations for wastewater treatment plants in South Africa – A realistic perspective. Water Institute of Southern Africa (WISA) Biannual Conference.

[bb0500] Schiemenz K., Eichler-Löbermann B. (2010). Biomass ashes and their phosphorus fertilizing effect on different crops. Nutr. Cycl. Agroecosyst..

[bb0505] Schönning C., Stenström T.A., Programme E. (2005). Guidelines for the safe use of urine and faeces in ecological sanitation systems. J. Indian Water Work. Assoc..

[bb0510] Schönning C., Westrell T., Stenström T.A., Arnbjerg-Nielsen K., Hasling A.B., Høibye L., Carlsen A. (2007). Microbial risk assessment of local handling and use of human faeces. J. Water Health.

[bb0520] Shanahan E.F., Roiko A., Tindale N.W., Thomas M.P., Walpole R., Ipek Kurtböke D. (2010). Evaluation of pathogen removal in a solar sludge drying facility using microbial indicators. Int. J. Environ. Res. Public Health.

[bb0525] Sibanda T., Okoh A.I. (2012). Assessment of the incidence of enteric adenovirus species and serotypes in surface waters in the eastern cape province of South Africa: Tyume River as a case study. ScientificWorldJournal.

[bb0530] Sobsey M., Guardabassi L., Dalsgaard A. (2003). Occurence and survival of viruses in composted human faeces. Sustain. Urban Renew. Wastewater Treat. Danish Environ. Prot. Agency.

[bb5005] Sommer S.G. (2001). Effect of composting on nutrient loss and nitrogen availability of cattle deep litter. Eur. J. Agron..

[bb0540] Stenström T.A., Seidu R., Nelson E., Christian Z. (2011). Microbial Exposure and Health Assessments in Sanitation Technologies and Systems.

[bb0545] Steyn M., Jagals P., Genthe B. (2004). Assessment of microbial infection risks posed by ingestion of water during domestic water use and full-contact recreation in a mid-southern African region. Water Sci. Technol..

[bb0550] The Nano Membrane Toilet http://www.nanomembranetoilet.org.

[bb0555] Thibodeau C., Monette F., Bulle C., Glaus M. (2014). Comparison of black water source-separation and conventional sanitation systems using life cycle assessment. J. Clean. Prod..

[bb0560] Tilley E., Lüthi C., Morel A., Zurbrügg C., Schertenleib R. (2014). Compendium of sanitation systems and technologies. Development.

[bb0565] Tsinda A., Abbott P., Pedley S., Charles K., Adogo J., Okurut K., Chenoweth J. (2013). Challenges to achieving sustainable sanitation in informal settlements of Kigali, Rwanda. Int. J. Environ. Res. Public Health.

[bb0570] UNICEF, WHO (2017). Progress on Drinking Water, Sanitation and Hygiene.

[bb0575] United Nations (2017). Progress towards the Sustainable Development Goals.

[bb0580] Van Abel N., Schoen M.E., Kissel J.C., Meschke J.S. (2017). Comparison of risk predicted by multiple norovirus dose–response models and implications for quantitative microbial risk assessment. Risk Anal..

[bb0585] Van Haaren R. (2009). Large Scale Aerobic Composting of Source ­ Separated Organic Wastes: A Comparative Study of Environmental Impacts, Costs, and Contextual Effects.

[bb0590] Viljoen N. (2015). City of Cape Town Residential Water Consumption Trend Analysis 2014/2015.

[bb0595] Wang H., Wang T., Zhang B., Li F., Toure B., Omosa I.B., Chiramba T., Abdel-Monem M., Pradhan M. (2014). Water and wastewater treatment in Africa - current practices and challenges. Clean: Soil, Air, Water.

[bb0605] Weidema B.P., Wesnaes M.S. (1996). Data quality management for life cycle inventories-an example of using data quality indicators. J. Clean. Prod..

[bb0610] WHO (2010). Health-Based Targets.

[bb0615] WHO (2016). Quantitative Microbial Risk Assessment: Application for Water Safety Management.

[bb0620] Winblad U., Simpson-Hébert M. (2004). Ecological Sanitation (Revised and Enlarged Edition).

