# Supplementary material for: Conceptual environmental impact assessment of a novel self-sustained sanitation system incorporating a quantitative microbial risk assessment approach
Source: Sci Total Environ. 2018 Oct 15;639:657–72. doi: 10.1016/j.scitotenv.2018.05.062 (PMC6021597; doi:10.1016/j.scitotenv.2018.05.062)
Supplement: Supplementary file 1 — Supplementary material [file mmc1.docx]

**Table S1. Inventory data employed in the LCA study**

| **Material/Energy Input** | **Inventory Data** |
| --- | --- |
| Polystyrene | - Polystyrene, general purpose {RoW}\| production \| Alloc Def, S |
| Sanitary ceramics (toilet bowl) | - Sanitary ceramics {RoW}\| production \| Alloc Def, S |
| Alloy Steel | - Steel, low-alloyed {RoW}\| steel production, converter, low-alloyed \| Alloc Def, S |
| Injection moulding | - Injection moulding {RoW}\| processing \| Alloc Def, S |
| Membrane | - Glass fibre reinforced plastic, polyamide, injection moulded {RoW}\| production \| Alloc Def, S |
| Water, Treated wastewater | - Tap water {RoW}\| tap water production, conventional treatment \| Alloc Def, S |
| Electricity | - Electricity, medium voltage {RoW}\| market for \| Alloc Def, S |
| Diesel | - Diesel, low-sulfur {RoW}\| production \| Alloc Def, S |
| Lime | - Lime, hydraulic {RoW}\| production \| Alloc Def, S |
| Iron chloride | - Iron(III) chloride, without water, in 14% iron solution state {RoW}\| production \| Alloc Def, S |
| Polymer | - Polyacrylamide {GLO}\| production \| Alloc Def, S |
| Transportation | - Transport, freight, lorry 16-32 metric ton, EURO6 {RoW}\| transport, freight, lorry 16-32 metric ton, EURO6 \| Alloc Def, S |
| N-Fertilizer | - Nitrogen fertiliser, as N {GLO}\| market for \| Alloc Def, S |
| P-Fertilizer | - Phosphate fertiliser, as P2O5 {GLO}\| market for \| Alloc Def, S |
| K-Fertilizer | - Potassium fertiliser, as K2O {GLO}\| market for \| Alloc Def, S |
| N_2_O | - Dinitrogen monoxide;low. pop., long-term |
| CH_4_ | - Methane; low. pop., long-term |
| NH_3_ | - Ammonia; low. pop., long-term |
| NO_X_ | - Nitrogen oxides; low. pop., long-term |

{ }: denotes the geographical location which the specific dataset refers to; {RoW} = Rest-of-the-World; Alloc Def, S = allocation, default-system; {GLO} = global; low.pop. = low density population

| **Parameter(or Variable)** | **Unit** | **Distribution** |
| --- | --- | --- |
| **Concentration of pathogens in faecal sludge (Cpathogen)-Route 1** | | |
| Enterotoxigenic E.coli (C_coli_) | Log_10_CFU/ ml | Uniform (5,12;6.12) |
| Shigella SPP. (C_Shig._) | Log_10_CFU/ ml | Uniform (2.12;3.12) |
| Cryptosporidium SPP(C_Crypto_.) | Log_10_CFU/ ml | Uniform (1.12;5.12) |
| Norovirus (C_Norov._) | Log_10_CFU/ ml | Uniform (5.12;6.12) |
| Rotavirus (C_Rotav_.) | Log_10_CFU/ ml | Uniform (3.12;8.12) |
| **Concentration of pathogens in river water (Cpathogen)-Route 2 & 3** | | |
| Enterotoxigenic E.coli (C_coli_) | Log_10_CFU/ ml | Uniform (2.53;3.53) |
| Shigella SPP. (C_Shig._) | Log_10_CFU/ ml | Uniform (-0.47;0.53) |
| Cryptosporidium SPP(C_Crypto_.) | Log_10_CFU/ ml | Uniform(-1.47;2.53) |
| Norovirus (C_Norov._) | Log_10_CFU/ ml | Uniform (2.53;3.53) |
| Rotavirus (C_Rotav_.) | Log_10_CFU/ ml | Uniform (0.53;5.53) |
| **Concentration of pathogens in faecal matter (Cpathogen)-Route 4** | | |
| Enterotoxigenic E.coli (C_coli_) | CFU/mg | Uniform (1.00E+04;1.00E+05) |
| Shigella SPP. (C_Shig._) | CFU/mg | Uniform (1.00E+01;1.00E+02) |
| Cryptosporidium SPP(C_Crypto_.) | CFU/mg | Uniform (1.00E+00;1.00E+04) |
| Norovirus (C_Norov._) | CFU/mg | Uniform (1.00E+04;1.00E+05) |
| Rotavirus (C_Rotav_.) | CFU/mg | Uniform (1.00E+02;1.00E+07) |

**Table S2. Input Parameters of Monte Carlo Simulations for QMRA analysis (Part 1)**

**Table S3. Input Parameters of Monte Carlo Simulations for QMRA analysis (Part 2)**

| **Parameter(or Variable)** | **Unit** | **Distribution** |
| --- | --- | --- |
| **Dose-Exposure** | |  |
| *Route 1* |  |  |
| Volume ingested (V_water_) | ml | Point Estimate: 5 (Labite et al., 2010) |
| Frequency (*f_exp_)* |  | Point estimate : 1 |
| *Route 2* |  |  |
| Volume ingested (V_water_) | ml | Uniform (10;100) (Steyn et al., 2004) |
| Frequency (f_exp_) |  | Point estimate : 1 |
| *Route 3* |  |  |
| Volume ingested (V_water_) | ml | Uniform (630;952) (Steyn et al., 2004) |
| Frequency (f_exp_) |  | Point estimate : 1 |
| *Route 4* |  |  |
| Volume ingested (V_water_) | mg | Uniform (8; 134) (Schönning et al., 2007) |
| Frequency (f_exp_) |  | Point estimate : 1 |
| **Dose-Response** | |  |
| Enterotoxigenic E.coli |  | Point estimate : α= 1.95E-01; N50=3.01E+07 (Mena et al., 2004) |
| Shigella SPP. |  | Point estimate : α=2.65E-01; N50=1.48E+03 (QMRAwiki, 2017) |
| Cryptosporidium SPP |  | Point estimate: r=4.19E-03 (QMRAwiki, 2017) |
| Norovirus |  | Point estimate: α= 4.00E-02; β=5.50E-02 (Van Abel et al., 2017) |
| Rotavirus |  | Point estimate: α=2.53E-01; N50=6.17E+00 (QMRAwiki, 2017) |
|  |  |  |
| **Risk Characterization** |  |  |
| Illness to infection ratio-P(ill\|inf) |  |  |
| Enterotoxigenic E.coli |  | Point estimate: 0.28 (Soller et al., 2010) |
| Shigella SPP. |  | Point estimate: 0.059 (Boveé et al., 2012) |
| Cryptosporidium SPP |  | Point estimate: 0.5 (Soller et al., 2010) |
| Norovirus |  | Point estimate: 0.67 (Kay, 2015) |
| Rotavirus |  | Point estimate: 0.05 (Havelaar and Melse, 2003) |
| DALYs per case of disease (BOD_PATHOGEN_) |  |  |
| Enterotoxigenic E.coli | DALYs/case | Point estimate: 1.09E-03 (Murray et al., 2012) |
| Shigella SPP. | DALYs/case | Point estimate: 1.02E-03 (Murray et al., 2012) |
| Cryptosporidium SPP | DALYs/case | Point estimate: 1.22E-03 (Murray et al., 2012) |
| Norovirus | DALYs/case | Point estimate: 8.00E-04 (Fuhrimann et al., 2016) |
| Rotavirus | DALYs/case | Point estimate: 2.71E-03 (Murray et al., 2012) |

| Process | Human Health  (DALY) | % | Ecosystems  (Species.Yr) | % | Resources  ($) | % |
| --- | --- | --- | --- | --- | --- | --- |
| Alloy steel | 6.25E-09 | 0.7% | 1.54E-11 | 0.9% | 2.92E-04 | 4.7% |
| Glass fibre-raw material | 2.32E-10 | 0.0% | 1.07E-12 | 0.1% | 6.68E-06 | 0.1% |
| Polystyrene | 1.49E-08 | 1.6% | 7.17E-11 | 4.3% | 8.01E-04 | 12.9% |
| Injection moulding | 1.21E-08 | 1.3% | 5.15E-11 | 3.1% | 2.59E-04 | 4.2% |
| Sanitary ceramics | 5.04E-08 | 5.5% | 1.36E-10 | 8.1% | 7.77E-04 | 12.5% |
| Transportation-Toilet | 3.59E-09 | 0.4% | 1.83E-11 | 1.1% | 1.13E-04 | 1.8% |
| Glass fibre-maintenance | 9.88E-09 | 1.1% | 4.58E-11 | 2.7% | 2.85E-04 | 4.6% |
| Electricity | -5.64E-08 | 6.1% | -2.64E-10 | 15.7% | -1.41E-03 | 22.7% |
| NOx emissions | 6.30E-07 | 68.2% | 3.58E-11 | 2.1% | - | 0.0% |
| Treated wastewater | -1.12E-08 | 1.2% | -3.99E-11 | 2.4% | -1.96E-04 | 3.2% |
| P-fertilizer | -7.36E-08 | 8.0% | -3.25E-10 | 19.3% | -1.41E-03 | 22.7% |
| K-fertilizer | -5.41E-08 | 5.9% | -6.75E-10 | 40.1% | -6.29E-04 | 10.1% |
| Transportation-Product | 8.80E-10 | 0.1% | 4.48E-12 | 0.3% | 2.78E-05 | 0.4% |

**Table S4. Contribution of the unit processes comprising the NMT system to the Human Health, Ecosystems and Resources Impact Categories**

| Process | Human Health  (DALY) | % | Ecosystems  (Species.Yr) | % | Resources  ($) | % |
| --- | --- | --- | --- | --- | --- | --- |
| Polystyrene | 2.11E-09 | 0.1% | 1.01E-11 | 0.1% | 1.13E-04 | 0.3% |
| Injection moulding | 1.32E-09 | 0.0% | 5.65E-12 | 0.0% | 2.83E-05 | 0.1% |
| Sanitary ceramics | 1.96E-08 | 0.7% | 5.30E-11 | 0.3% | 3.02E-04 | 0.8% |
| Transportation-Toilet | 1.11E-09 | 0.0% | 5.65E-12 | 0.0% | 3.50E-05 | 0.1% |
| Transportation- Waste Management | 1.62E-08 | 0.5% | 8.23E-11 | 0.5% | 5.11E-04 | 1.4% |
| Diesel | 1.46E-08 | 0.5% | 8.19E-11 | 0.5% | 2.39E-03 | 6.4% |
| Electricity | 7.62E-09 | 0.3% | 3.56E-11 | 0.2% | 1.90E-04 | 0.5% |
| CH4 emissions | 2.80E-07 | 9.5% | 1.58E-09 | 9.2% | - | 0.0% |
| NH3 emissions | 1.18E-07 | 4.0% | 2.02E-11 | 0.1% | - | 0.0% |
| N2O emissions | 2.00E-07 | 6.8% | 1.13E-09 | 6.6% | - | 0.0% |
| N-fertilizer | -1.85E-06 | 62.6% | -1.06E-08 | 61.6% | -2.47E-02 | 66.2% |
| P-fertilizer | -9.42E-08 | 3.2% | -4.16E-10 | 2.4% | -1.80E-03 | 4.8% |
| K-fertilizer | -1.92E-07 | 6.5% | -2.40E-09 | 13.9% | -2.24E-03 | 6.0% |
| Transportation-Product | 1.59E-07 | 5.4% | 8.07E-10 | 4.7% | 5.00E-03 | 13.4% |

**Table S5. Contribution of the unit processes comprising the UDDT system to the Human Health, Ecosystems and Resources Impact Categories**

**Table S6. Contribution of the unit processes comprising the PFT system to the Human Health, Ecosystems and Resources Impact Categories**

| Process | Human Health  (DALYs) | % | Ecosystems  (Species.Yr) | % | Resources  ($) | % |
| --- | --- | --- | --- | --- | --- | --- |
| Polystyrene | 2.11E-09 | 0.1% | 1.0E-11 | 0.1% | 1.13E-04 | 0.2% |
| Injection moulding | 1.32E-09 | 0.1% | 5.6E-12 | 0.1% | 2.83E-05 | 0.1% |
| Sanitary ceramics | 1.96E-08 | 0.9% | 5.3E-11 | 0.5% | 3.02E-04 | 0.6% |
| Transportation-Toilet | 1.11E-09 | 0.1% | 5.6E-12 | 0.1% | 3.50E-05 | 0.1% |
| Transportation- Waste Management | 1.24E-06 | 57.5% | 6.3E-09 | 55.8% | 3.90E-02 | 80.2% |
| Polymer | 1.18E-10 | 0.0% | 5.0E-13 | 0.0% | 5.31E-06 | 0.0% |
| FeCl_3_ | 2.58E-11 | 0.0% | 8.7E-14 | 0.0% | 4.79E-07 | 0.0% |
| Lime | 3.10E-09 | 0.1% | 1.5E-11 | 0.1% | 3.60E-05 | 0.1% |
| Electricity | 8.30E-08 | 3.9% | 3.9E-10 | 3.4% | 2.07E-03 | 4.3% |
| N2O emissions | 3.05E-07 | 14.2% | 1.7E-09 | 15.3% |  | 0.0% |
| N-fertilizer | -4.38E-07 | 20.4% | -2.5E-09 | 22.3% | -5.85E-03 | 12.0% |
| P-fertilizer | -6.11E-08 | 2.8% | -2.7E-10 | 2.4% | -1.17E-03 | 2.4% |
| Transportation-Product | 1.17E-10 | 0.0% | 5.9E-13 | 0.0% | 3.68E-06 | 0.0% |

**Table S7. The minimum (Min), maximum (Max), mean, median and standard deviation (STD) values of the pathogen concentration for each exposure route as estimated by the Monte Carlo simulations- Base Case Scenario**

| Pathogen | Min | Max | Mean | Median | STD |
| --- | --- | --- | --- | --- | --- |
| **Route 1** | | | | | |
| Enterotoxigenic E.coli | 3.1530e-05 | 1.0411e-04 | 7.8502e-05 | 8.2898e-05 | 1.9485e-05 |
| Shigella SPP. | 3.1530e-05 | 1.0411e-04 | 7.8502e-05 | 8.2898e-05 | 1.9485e-05 |
| Cryptosporidium SPP. | 2.3042e-04 | 6.1000e-04 | 6.0978e-04 | 6.1000e-04 | 7.0973e-06 |
| Norovirus | 2.2250e-04 | 2.5007e-04 | 2.4145e-04 | 2.4320e-04 | 6.9066e-06 |
| Rotavirus | 1.2938e-04 | 1.3486e-04 | 1.3464e-04 | 1.3474e-04 | 3.1259e-07 |
| **Route 2** | | | | | |
| Enterotoxigenic E.coli | 2.6637e-07 | 1.8423e-05 | 6.2266e-06 | 5.3249e-06 | 4.1163e-06 |
| Shigella SPP. | 4.8504e-07 | 1.8053e-05 | 8.3758e-06 | 8.0712e-06 | 4.2262e-06 |
| Cryptosporidium SPP. | 5.3071e-06 | 6.1000e-04 | 5.9901e-04 | 6.1000e-04 | 5.7326e-05 |
| Norovirus | 1.4946e-04 | 2.1373e-04 | 1.9378e-04 | 1.9555e-04 | 1.1506e-05 |
| Rotavirus | 1.1876e-04 | 1.3413e-04 | 1.3327e-04 | 1.3351e-04 | 8.7553e-07 |
| **Route 3** | | | | | |
|  |  |  |  |  |  |
| Enterotoxigenic E.coli | 1.2649e-05 | 7.8419e-05 | 4.9853e-05 | 5.2572e-05 | 1.6247e-05 |
| Shigella SPP. | 1.4561e-05 | 3.5358e-05 | 2.8665e-05 | 2.9798e-05 | 4.5643e-06 |
| Cryptosporidium SPP. | 9.0712e-05 | 6.1000e-04 | 6.0957e-04 | 6.1000e-04 | 1.0336e-05 |
| Norovirus | 2.0804e-04 | 2.4148e-04 | 2.3030e-04 | 2.3206e-04 | 7.3178e-06 |
| Rotavirus | 1.2626e-04 | 1.3473e-04 | 1.3442e-04 | 1.3454e-04 | 3.8151e-07 |
| **Route 4** | | | | | |
| Enterotoxigenic E.coli | 5.7653e-06 | 1.2684e-04 | 7.5989e-05 | 7.8534e-05 | 2.8230e-05 |
| Shigella SPP. | 8.0010e-06 | 4.3016e-05 | 3.4065e-05 | 3.5401e-05 | 6.1055e-06 |
| Cryptosporidium SPP. | 1.6789e-04 | 6.1000e-04 | 6.0969e-04 | 6.1000e-04 | 9.3330e-06 |
| Norovirus | 1.9723e-04 | 2.5794e-04 | 2.3983e-04 | 2.4136e-04 | 1.0670e-05 |
| Rotavirus | 1.2873e-04 | 1.3496e-04 | 1.3461e-04 | 1.3470e-04 | 3.5225e-07 |

| Pathogen | Min | Max | Mean | Median | STD |
| --- | --- | --- | --- | --- | --- |
| **Route 1** | | | | | |
| Enterotoxigenic E.coli | 1.2842e-04 | 2.6730e-04 | 2.3143e-04 | 2.4291e-04 | 3.3929e-05 |
| Shigella SPP. | 5.5337e-05 | 5.9902e-05 | 5.9196e-05 | 5.9582e-05 | 9.2572e-07 |
| Cryptosporidium SPP. | 6.0752e-04 | 6.1000e-04 | 6.1000e-04 | 6.1000e-04 | 2.6785e-08 |
| Norovirus | 4.9931e-04 | 5.1285e-04 | 5.0898e-04 | 5.0993e-04 | 3.2846e-06 |
| Rotavirus | 1.3550e-04 | 1.3550e-04 | 1.3550e-04 | 1.3550e-04 | 4.2297e-13 |
| **Route 2** | | | | | |
| Enterotoxigenic E.coli | 1.2027e-06 | 8.0780e-05 | 2.9512e-05 | 2.6042e-05 | 1.8576e-05 |
| Shigella SPP. | 2.3325e-06 | 4.9977e-05 | 2.9607e-05 | 3.0617e-05 | 1.1662e-05 |
| Cryptosporidium SPP. | 2.3408e-05 | 6.1000e-04 | 6.0782e-04 | 6.1000e-04 | 6.1000e-04 |
| Norovirus | 4.3177e-04 | 4.9380e-04 | 4.7853e-04 | 4.8030e-04 | 9.9957e-06 |
| Rotavirus | 1.3542e-04 | 1.3550e-04 | 1.3550e-04 | 1.3550e-04 | 8.0160e-10 |
| **Route 3** | | | | | |
| Enterotoxigenic E.coli | 5.7541e-05 | 2.3557e-04 | 1.7474e-04 | 1.8558e-04 | 4.2641e-05 |
| Shigella SPP. | 4.5189e-05 | 5.9463e-05 | 5.7232e-05 | 5.8250e-05 | 2.5251e-06 |
| Cryptosporidium SPP. | 4.1200e-04 | 6.1000e-04 | 6.0994e-04 | 6.1000e-04 | 2.7199e-06 |
| Norovirus | 4.9010e-04 | 5.0919e-04 | 5.0342e-04 | 5.0454e-04 | 4.0684e-06 |
| Rotavirus | 1.3550e-04 | 1.3550e-04 | 1.3550e-04 | 1.3550e-04 | 5.9563e-13 |
| **Route 4** | | | | | |
| Enterotoxigenic E.coli | 2.8189e-05 | 2.8473e-04 | 2.1959e-04 | 2.3514e-04 | 5.2604e-05 |
| Shigella SPP. | 3.2415e-05 | 6.0069e-05 | 5.8584e-05 | 5.9456e-05 | 2.4047e-06 |
| Cryptosporidium SPP. | 4.5772e-04 | 6.1000e-04 | 6.0998e-04 | 6.1000e-04 | 1.5425e-06 |
| Norovirus | 4.8227e-04 | 5.1583e-04 | 5.0797e-04 | 5.0913e-04 | 5.3935e-06 |
| Rotavirus | 1.3550e-04 | 1.3550e-04 | 1.3550e-04 | 1.3550e-04 | 4.5693e-13 |

**Table S8. The minimum (Min), maximum (Max), mean, median and standard deviation (STD) values of the pathogen concentration for each exposure route as estimated by the Monte Carlo simulations- fexp=5**

**Table S9. The minimum (Min), maximum (Max), mean, median and standard deviation (STD) values of the pathogen concentration for each exposure route as estimated by the Monte Carlo simulations- P(ill|inf)’= 1.2 P(ill|inf)**

| Pathogen | Min | Max | Mean | Median | STD |
| --- | --- | --- | --- | --- | --- |
| **Route 1** | | | | | |
| Enterotoxigenic E.coli | 3.7835e-05 | 1.2493e-04 | 9.4501e-05 | 1.0016e-04 | 2.3270e-05 |
| Shigella SPP. | 2.8589e-05 | 4.7588e-05 | 4.2081e-05 | 4.3519e-05 | 4.7330e-06 |
| Cryptosporidium SPP. | 4.9775e-04 | 7.3200e-04 | 7.3191e-04 | 7.3200e-04 | 4.0009e-06 |
| Norovirus | 2.6700e-04 | 3.0009e-04 | 2.8951e-04 | 2.9164e-04 | 8.4270e-06 |
| Rotavirus | 1.5360e-04 | 1.6183e-04 | 1.6158e-04 | 1.6169e-04 | 3.4584e-07 |
| **Route 2** | | | | | |
| Enterotoxigenic E.coli | 3.0844e-07 | 2.2026e-05 | 7.4730e-06 | 6.4172e-06 | 4.9050e-06 |
| Shigella SPP. | 6.1983e-07 | 2.1695e-05 | 1.0051e-05 | 9.6280e-06 | 5.0855e-06 |
| Cryptosporidium SPP. | 6.5057e-06 | 7.3200e-04 | 7.1750e-04 | 7.3200e-04 | 7.3055e-05 |
| Norovirus | 1.8103e-04 | 2.5629e-04 | 2.3276e-04 | 2.3449e-04 | 1.3620e-05 |
| Rotavirus | 1.4467e-04 | 1.6096e-04 | 1.5994e-04 | 1.6020e-04 | 9.9549e-07 |
| **Route 3** | | | | | |
| Enterotoxigenic E.coli | 1.5351e-05 | 9.3890e-05 | 5.9902e-05 | 6.2988e-05 | 1.9334e-05 |
| Shigella SPP. | 1.7404e-05 | 4.2446e-05 | 3.4360e-05 | 3.5795e-05 | 5.5517e-06 |
| Cryptosporidium SPP. | 9.3511e-05 | 7.3200e-04 | 7.3129e-04 | 7.3200e-04 | 1.5582e-05 |
| Norovirus | 2.4949e-04 | 2.8970e-04 | 2.7626e-04 | 2.7842e-04 | 8.9801e-06 |
| Rotavirus | 1.5444e-04 | 1.6168e-04 | 1.6130e-04 | 1.6144e-04 | 4.5892e-07 |
| **Route 4** | | | | | |
| Enterotoxigenic E.coli | 7.3021e-06 | 1.5252e-04 | 9.0534e-05 | 9.3136e-05 | 3.3640e-05 |
| Shigella SPP. | 1.0298e-05 | 5.1655e-05 | 4.0917e-05 | 4.2546e-05 | 7.3654e-06 |
| Cryptosporidium SPP. | 2.3210e-04 | 7.3200e-04 | 7.3156e-04 | 7.3200e-04 | 1.1151e-05 |
| Norovirus | 2.3565e-04 | 3.0935e-04 | 2.8797e-04 | 2.8980e-04 | 1.2726e-05 |
| Rotavirus | 1.5624e-04 | 1.6196e-04 | 1.6153e-04 | 1.6164e-04 | 4.2037e-07 |

**Table S10. Pedigree matrix and uncertainty factors adopted from (Weidema and Beaufort, 2001) and (Ciroth et al., 2016), respectively.**

| **Indicator Score** | **1** | **2** | **3** | **4** | **5** |
| --- | --- | --- | --- | --- | --- |
| Reliabili**ty**  **(U1)** | Verified data based on  measurements | Verified data partly based on  assumptions or non-verified  data based on measurements | Non-verified data partly  based on qualified estimates | Qualified estimate  (e.g. by industrial expert) | Non-qualified estimate |
|  | 1 | 1.54 | 1.61 | 1.69 | n.a |
| **Completeness (U2)** | Representative data from all  sited relevant for the market  considered, over an adequate  period even out normal  fluctuations | Representative data from >50 %  of the sites relevant for the market  considered, over an adequate period  to even out normal fluctuations | Representative data from only  some sited (≪50 %) relevant  for the market considered or  >50 % of sites but from  shorter periods | Representative data from only  one site relevant for the market  considered or some sites but  from shorter periods | Representativeness  unknown or data  from a small number  of sites and from  shorter periods |
|  | 1 | 1.03 | 1.04 | 1.08 | n.a |
| **Temporal Correlation**  **(U3)** | Less than 3 years of  difference to the time  period of the dataset | Less than 6 years of difference  of the time period of the dataset | Less than 10 years of  difference to the time period  of the dataset | Less than 15 years of difference  to the time period of the dataset | Age of data unknown or  more than 15 years of  difference to the time  period of the dataset |
|  | 1 | 1.03 | 1.1 | 1.19 | 1.29 |
| **Geographical Correlation**  **(U4)** | Data from area under study | Average data from larger area  in which the area under  study is included | Data from area with similar  production conditions | Data from area with slightly  similar production conditions | Data from unknown or  distinctly different  area (North America  instead of Middle  East, OECD-Europe  instead of Russia) |
|  | 1 | 1.04 | 1.08 | 1.11 | n.a |
| **Further Technological Correlation**  **(U5)** | Data from enterprises,  processes and materials  under study | Data from processes and materials  under study (i.e. identical technology)  but from different enterprises | Data from processes and  materials under study from  different technology | Data on related processes  or materials | Data on related processes  on laboratory scale  or from different  technology |
|  | 1 | 1.18 | 1.65 | 2.08 | 2.8 |

**Table S11. Data quality assessment for all foreground data employed in the LCA study**

| **Life Cycle Phase** | **Input** | **U1** | **U2** | **U3** | **U4** | **U5** | **Ub*** | **SD^2** |
| --- | --- | --- | --- | --- | --- | --- | --- | --- |
| **Manufacture** | Polystyrene | 2 | 3 | 2 | 3 | 2 | 1.05 | 1.61 |
|  | Alloy Steel | 2 | **4** | 1 | 3 | 2 | 1.05 | 1.61 |
|  | Sanitary ceramics (toilet bowl) | 2 | 3 | 2 | 3 | 2 | 1.05 | 1.61 |
|  | Injection moulding | 2 | 4 | 2 | 3 | 2 | 1.05 | 1.61 |
|  | Transportation | 2 | 4 | 1 | 3 | 2 | 2.00 | 2.32 |
| **Use/Maintenance** | Membrane (glass fibre) | 2 | **4** | 1 | 3 | 2 | 1.05 | 1.61 |
|  | Urine | 1 | 2 | 1 | 2 | 1 | 1.05 | 1.07 |
|  | Faeces | 1 | 2 | 1 | 2 | 1 | 1.05 | 1.07 |
|  | Water | 1 | 2 | 1 | 2 | 1 | 1.05 | 1.07 |
| **Waste Management** | Lime | 1 | 3 | 4 | 3 | 2 | 1.05 | 1.30 |
|  | Electricity (NMT) | 2 | 4 | 1 | 3 | 2 | 1.05 | 1.61 |
|  | Electricity (Other systems) | 1 | 3 | 4 | 3 | 2 | 1.05 | 1.30 |
|  | Polymer | 1 | 3 | 4 | 3 | 2 | 1.05 | 1.30 |
|  | Diesel | 1 | 3 | 4 | 3 | 2 | 1.05 | 1.30 |
|  | FeCl_3_ | 1 | 3 | 4 | 3 | 2 | 1.05 | 1.30 |
|  | NH_3_ emissions | 1 | 2 | 4 | 3 | 2 | 1.2 | 1.37 |
|  | CH_4_ emissions | 1 | 2 | 4 | 3 | 2 | 1.2 | 1.37 |
|  | N_2_O emissions | 1 | 2 | 4 | 3 | 2 | 1.4 | 1.52 |
| **Product** | Treated wastewater | 2 | 4 | 1 | 3 | 2 | 1.05 | 1.61 |
|  | Electricity | 2 | 4 | 1 | 3 | 2 | 1.05 | 1.61 |
|  | NOx emissions | 2 | 4 | 1 | 3 | 2 | 1.50 | 1.87 |
|  | N-fertilizer | 2 | 4 | 2 | 3 | 2 | 1.05 | 1.61 |
|  | K-fertilizer | 2 | 4 | 2 | 3 | 2 | 1.05 | 1.61 |
|  | P-Fertilizer | 2 | 4 | 2 | 3 | 2 | 1.05 | 1.61 |
|  | Transportation | 2 | 4 | 1 | 3 | 2 | 2.00 | 2.32 |

*The uncertainty factor Ub has been adopted from (Frischknecht et al., 2007).

REFERENCES

Boveé, L., Whelan, J., Sonder, G.J., van Dam, A.P., van den Hoek, A., Risk factors for secondary transmission of Shigellainfection within households: implications for current prevention policy. *BMC Infect. Dis.* **12**, 2012, 347. https://doi.org/10.1186/1471-2334-12-347

Ciroth, A., Muller, S., Weidema, B., Lesage, P., Empirically based uncertainty factors for the pedigree matrix in ecoinvent. *Int. J. Life Cycle Assess.* **21**, 2016, 1338–1348. https://doi.org/10.1007/s11367-013-0670-5

Frischknecht, R., Jungbluth, N., Althaus, H., Doka, G., Dones, R., Heck, T., Hellweg, S., Hischier, R., Nemecek, T., Rebitzer, G., Spielmann, M., Wernet, G., Overview and Methodology. Ecoinvent Centre, 2017, 1–77.

Fuhrimann, S., Winkler, M.S., Stalder, M., Niwagaba, C.B., Babu, M., Kabatereine, N.B., Halage, A.A., Utzinger, J., Cissé, G., Nauta, M., Disease burden due to gastrointestinal pathogens in a wastewater system in Kampala, Uganda. M*icrob. Risk Anal.* **4**, 2016, 16–28. https://doi.org/10.1016/j.mran.2016.11.003

Havelaar, A.H., Melse, J.M., Quantifying public health risk in the WHO Guidelines for Drinking Water Quality: a burden of disease approach. RIVM Rep. 734301022, 2003, 1–49.

Kay, D., Section Introduction: Investigative Tools, in Routledge Handbook of Water and Health. Routledge, 2015.

Labite, H., Lunani, I., Van Der Steen, P., Vairavamoorthy, K., Drechsel, P., Lens, P., Quantitative microbial risk analysis to evaluate health effects of interventions in the urban water system of Accra, Ghana. *J. Water Health* **8**, 2010, 417–430. https://doi.org/10.2166/wh.2010.021

Mena, K.D., Rose, J.B., Gerba, C.P., Addressing Microbial Food Safety Issues Quantitatively: A Risk Assessment Approach in Preharvest and Postharvest Food Safety: Contemporary Issues and Future Directions. 2004.

Murray, C.J.L., Vos, T., Lozano, R., Naghavi, M., Flaxman, A.D., Michaud, C., Ezzati, M., Shibuya, K., Salomon, J.A., Abdalla, S., Aboyans, V., Abraham, J., Ackerman, I., Aggarwal, R., Ahn, S.Y., Ali, M.K., Alvarado, M., Anderson, H.R., Anderson, L.M., Andrews, K.G., Atkinson, C., Baddour, L.M., Bahalim, A.N., Barker-Collo, S., Barrero, L.H., Bartels, D.H., Basáñez, M.G., Baxter, A., Bell, M.L., Benjamin, E.J., Bennett, D., Bernabé, E., Bhalla, K., Bhandari, B., Bikbov, B., Abdulhak, A. Bin, Birbeck, G., Black, J.A., Blencowe, H., Blore, J.D., Blyth, F., Bolliger, I., Bonaventure, A., Boufous, S., Bourne, R., Boussinesq, M., Braithwaite, T., Brayne, C., Bridgett, L., Brooker, S., Brooks, P., Brugha, T.S., Bryan-Hancock, C., Bucello, C., Buchbinder, R., Buckle, G., Budke, C.M., Burch, M., Burney, P., Burstein, R., Calabria, B., Campbell, B., Canter, C.E., Carabin, H., Carapetis, J., Carmona, L., Cella, C., Charlson, F., Chen, H., Cheng, A.T.A., Chou, D., Chugh, S.S., Coffeng, L.E., Colan, S.D., Colquhoun, S., Colson, K.E., Condon, J., Connor, M.D., Cooper, L.T., Corriere, M., Cortinovis, M., De Vaccaro, K.C., Couser, W., Cowie, B.C., Criqui, M.H., Cross, M., Dabhadkar, K.C., Dahiya, M., Dahodwala, N., Damsere-Derry, J., Danaei, G., Davis, A., De Leo, D., Degenhardt, L., Dellavalle, R., Delossantos, A., Denenberg, J., Derrett, S., Des Jarlais, D.C., Dharmaratne, S.D., Dherani, M., Diaz-Torne, C., Dolk, H., Dorsey, E.R., Driscoll, T., Duber, H., Ebel, B., Edmond, K., Elbaz, A., Ali, S.E., Erskine, H., Erwin, P.J., Espindola, P., Ewoigbokhan, S.E., Farzadfar, F., Feigin, V., Felson, D.T., Ferrari, A., Ferri, C.P., Fèvre, E.M., Finucane, M.M., Flaxman, S., Flood, L., Foreman, K., Forouzanfar, M.H., Fowkes, F.G.R., Fransen, M., Freeman, M.K., Gabbe, B.J., Gabriel, S.E., Gakidou, E., Ganatra, H.A., Garcia, B., Gaspari, F., Gillum, R.F., Gmel, G., Gonzalez-Medina, D., Gosselin, R., Grainger, R., Grant, B., Groeger, J., Guillemin, F., Gunnell, D., Gupta, R., Haagsma, J., Hagan, H., Halasa, Y.A., Hall, W., Haring, D., Haro, J.M., Harrison, J.E., Havmoeller, R., Hay, R.J., Higashi, H., Hill, C., Hoen, B., Hoffman, H., Hotez, P.J., Hoy, D., Huang, J.J., Ibeanusi, S.E., Jacobsen, K.H., James, S.L., Jarvis, D., Jasrasaria, R., Jayaraman, S., Johns, N., Jonas, J.B., Karthikeyan, G., Kassebaum, N., Kawakami, N., Keren, A., Khoo, J.P., King, C.H., Knowlton, L.M., Kobusingye, O., Koranteng, A., Krishnamurthi, R., Laden, F., Lalloo, R., Laslett, L.L., Lathlean, T., Leasher, J.L., Lee, Y.Y., Leigh, J., Levinson, D., Lim, S.S., Limb, E., Lin, J.K., Lipnick, M., Lipshultz, S.E., Liu, W., Loane, M., Ohno, S.L., Lyons, R., Mabweijano, J., MacIntyre, M.F., Malekzadeh, R., Mallinger, L., Manivannan, S., Marcenes, W., March, L., Margolis, D.J., Marks, G.B., Marks, R., Matsumori, A., Matzopoulos, R., Mayosi, B.M., McAnulty, J.H., McDermott, M.M., McGill, N., McGrath, J., Medina-Mora, M.E., Meltzer, M., Mensah, G.A., Merriman, T.R., Meyer, A.C., Miglioli, V., Miller, M., Miller, T.R., Mitchell, P.B., Mock, C., Mocumbi, A.O., Moffitt, T.E., Mokdad, A.A., Monasta, L., Montico, M., Moradi-Lakeh, M., Moran, A., Morawska, L., Mori, R., Murdoch, M.E., Mwaniki, M.K., Naidoo, K., Nair, M.N., Naldi, L., Narayan, K.M.V., Nelson, P.K., Nelson, R.G., Nevitt, M.C., Newton, C.R., Nolte, S., Norman, P., Norman, R., O’Donnell, M., O’Hanlon, S., Olives, C., Omer, S.B., Ortblad, K., Osborne, R., Ozgediz, D., Page, A., Pahari, B., Pandian, J.D., Rivero, A.P., Patten, S.B., Pearce, N., Padilla, R.P., Perez-Ruiz, F., Perico, N., Pesudovs, K., Phillips, D., Phillips, M.R., Pierce, K., Pion, S., Polanczyk, G. V., Polinder, S., Pope, C.A., Popova, S., Porrini, E., Pourmalek, F., Prince, M., Pullan, R.L., Ramaiah, K.D., Ranganathan, D., Razavi, H., Regan, M., Rehm, J.T., Rein, D.B., Remuzzi, G., Richardson, K., Rivara, F.P., Roberts, T., Robinson, C., De Leòn, F.R., Ronfani, L., Room, R., Rosenfeld, L.C., Rushton, L., Sacco, R.L., Saha, S., Sampson, U., Sanchez-Riera, L., Sanman, E., Schwebel, D.C., Scott, J.G., Segui-Gomez, M., Shahraz, S., Shepard, D.S., Shin, H., Shivakoti, R., Singh, D., Singh, G.M., Singh, J.A., Singleton, J., Sleet, D.A., Sliwa, K., Smith, E., Smith, J.L., Stapelberg, N.J.C., Steer, A., Steiner, T., Stolk, W.A., Stovner, L.J., Sudfeld, C., Syed, S., Tamburlini, G., Tavakkoli, M., Taylor, H.R., Taylor, J.A., Taylor, W.J., Thomas, B., Thomson, W.M., Thurston, G.D., Tleyjeh, I.M., Tonelli, M., Towbin, J.A., Truelsen, T., Tsilimbaris, M.K., Ubeda, C., Undurraga, E.A., Van Der Werf, M.J., Van Os, J., Vavilala, M.S., Venketasubramanian, N., Wang, M., Wang, W., Watt, K., Weatherall, D.J., Weinstock, M.A., Weintraub, R., Weisskopf, M.G., Weissman, M.M., White, R.A., Whiteford, H., Wiebe, N., Wiersma, S.T., Wilkinson, J.D., Williams, H.C., Williams, S.R.M., Witt, E., Wolfe, F., Woolf, A.D., Wulf, S., Yeh, P.H., Zaidi, A.K.M., Zheng, Z.J., Zonies, D., Lopez, A.D., Disability-adjusted life years (DALYs) for 291 diseases and injuries in 21 regions, 1990-2010: A systematic analysis for the Global Burden of Disease Study 2010. *The Lancet* **380**, 2012, 2197–2223. https://doi.org/10.1016/S0140-6736(12)61689-4

QMRAwiki, Dose response assessment [WWW Document]. URL http://qmrawiki.canr.msu.edu/index.php/Dose_response_assessment, 2017.

Schönning, C., Westrell, T., Stenström, T.A., Arnbjerg-Nielsen, K., Hasling, A.B., Høibye, L., Carlsen, A., Microbial risk assessment of local handling and use of human faeces. *J. Water Health* **5**, 2007,117–128. https://doi.org/10.2166/wh.2006.049

Soller, J.A., Schoen, M.E., Bartrand, T., Ravenscroft, J.E., Ashbolt, N.J., Estimated human health risks from exposure to recreational waters impacted by human and non-human sources of faecal contamination. Water Res. 44, 2010, 4674–4691. https://doi.org/10.1016/j.watres.2010.06.049

Steyn, M., Jagals, P., Genthe, B., Assessment of microbial infection risks posed by ingestion of water during domestic water use and full-contact recreation in a mid-southern African region. *Water Sci. Technol.* **50**, 2004, 301–308.

Van Abel, N., Schoen, M.E., Kissel, J.C., Meschke, J.S., Comparison of Risk Predicted by Multiple Norovirus Dose–Response Models and Implications for Quantitative Microbial Risk Assessment. *Risk Anal.* **37**, 2017, 245–264. https://doi.org/10.1111/risa.12616

Weidema, B., Beaufort, A.S.H. De, Framework for Modelling Data Uncertainty in Life Cycle Inventories. Int. *J. Life Cycle Assess.* **6**, 2001, 127–132. https://doi.org/10.1007/BF02978728
